# Supplementary material for: The WHEAT pilot trial—WithHolding Enteral feeds Around packed red cell Transfusion to prevent necrotising enterocolitis in preterm neonates: a multicentre, electronic patient record (EPR), randomised controlled point-of-care pilot trial
Source: BMJ Open. 2019 Sep 20;9(9):e033543. doi: 10.1136/bmjopen-2019-033543 (PMC6756449; doi:10.1136/bmjopen-2019-033543)
Supplement: Author's manuscript [file bmjopen-2019-033543.draft_revisions.pdf]

BMJ Open

BMJ Open is committed to open peer review. As part of this commitment we make the peer review history of every article we publish publicly available.

When an article is published we post the peer reviewers' comments and the authors' responses online. We also post the versions of the paper that were used during peer review. These are the versions that the peer review comments apply to.

The versions of the paper that follow are the versions that were submitted during the peer review process. They are not the versions of record or the final published versions. They should not be cited or distributed as the published version of this manuscript.

BMJ Open is an open access journal and the full, final, typeset and author-corrected version of record of the manuscript is available on our site with no access controls, subscription charges or pay-per-view fees (http://bmjopen.bmj.com).

If you have any questions on BMJ Open's open peer review process please email info.bmjopen@bmj.com

Study Protocol: The WHEAT pilot trial - WithHolding Enteral feeds Around packed red cell Transfusion to prevent necrotising enterocolitis in preterm neonates: a multi-centre, electronic patient record (EPR), randomised controlled point-of-care pilot trial

Journal:	*BMJ Open*
Manuscript ID	bmjopen-2019-033543
Article Type:	Protocol
Date Submitted by the Author:	12-Aug-2019
Complete List of Authors:	Gale, Chris; Imperial College London, Neonatal Medicine, School of Public Health, Chelsea and Westminster campus<br>Modi, Neena; Imperial College London, Neonatal Medicine, School of Public Health, Chelsea and Westminster campus<br>Jawad, Sena; Imperial College London, Neonatal Data Analysis Unit, School of Public Health, Chelsea and Westminster campus<br>Culshaw, Lucy; Bliss – The National Charity for the Newborn<br>Dorling, Jon; Dalhousie University, IWK Health Centre, Division of Neonatal-Perinatal Medicine, Faculty of Medicine<br>Bowler, Ursula; National Perinatal Epidemiology Unit, Clinical Trials Unit, Nuffield Department of Population Health, University of Oxford, Old Road Campus<br>Forster, Amanda; James Cook University Hospital, Neonatal Uni<br>King, Andy; National Perinatal Epidemiology Unit, Clinical Trials Unit, Nuffield Department of Population Health, University of Oxford, Old Road Campus<br>McLeish, Jenny; National Perinatal Epidemiology Unit, Nuffield Department of Population Health, University of Oxford, Old Road Campus<br>Linsell, Louise; National Perinatal Epidemiology Unit, Clinical Trials Unit, Nuffield Department of Population Health, University of Oxford, Old Road Campus<br>Turner, Mark; University of Liverpool, Women's and Children's Health, Institute of Translational Medicine<br>Robberts, Helen; Bliss – The National Charity for the Newborn, Parent of preterm twins<br>Stanbury, Kayleigh; National Perinatal Epidemiology Unit, Clinical Trials Unit, Nuffield Department of Population Health, University of Oxford, Old Road Campus<br>van Staa, Tjeerd; The University of Manchester, Centre for Health Informatics, Division of Informatics, Imaging and Data Science, School of Health Sciences, Faculty of Biology, Medicine and Health<br>Juszczak, Ed; National Perinatal Epidemiology Unit, Clinical Trials Unit, Nuffield Department of Population Health, University of Oxford, Old Road Campus
Keywords:	randomised controlled trial, preterm infant, Blood bank & transfusion medicine < HAEMATOLOGY, NEONATOLOGY, NNRD, electronic patient

	records

SCHOLARONE™
Manuscripts

Title page

Study Protocol: The WHEAT pilot trial - WithHolding Enteral feeds Around packed red cell Transfusion to prevent necrotising enterocolitis in preterm neonates: a multi-centre, electronic patient record (EPR), randomised controlled point-of-care pilot trial

Gale C[1], Modi N[1], Jawad S[1], Culshaw L[2], Dorling J[3], Bowler U[4], Forster A[5], King A[4], McLeish J[4], Linsell L[4], Turner M[6], Robberts H[7], Stanbury K[4], van Staa TP[8], Juszczak E[4]

Institutions

[1] Neonatal Data Analysis Unit, Imperial College London, UK

[2] Bliss – The National Charity for the Newborn, Chapter House, 18-20 Crucifix Lane, London, SE1 3JW

[3] Division of Neonatal-Perinatal Medicine, IWK Health Centre, Dalhousie University, Halifax, NS, Canada B3H 4R2.

[4] National Perinatal Epidemiology Unit, Nuffield Department of Population Health, University of Oxford

[5] Research Nurse, Neonatal Unit, James Cook University Hospital, Marton Road, Middlesbrough, TS4 3BW

[6] Professor of Neonatology and Research Delivery, Women's and Children's Health, Institute of Translational Medicine, University of Liverpool, Liverpool, L69 3BX

[7] Parent of preterm twins, c/o Bliss – The National Charity for the Newborn, Chapter House, 18-20 Crucifix Lane, London, SE1 3JW

For peer review only - http://bmjopen.bmj.com/site/about/guidelines.xhtml

[8] Professor in Health e-Research, Centre for Health Informatics, Division of Informatics, Imaging and Data Science, School of Health Sciences, Faculty of Biology, Medicine and Health, The University of Manchester, Manchester Academic Health Science Centre, Manchester, United Kingdom

Authors

Neena Modi n.modi@imperial.ac.uk, Neonatal Data Analysis Unit, Imperial College London, Chelsea and Westminster Hospital campus, 369 Fulham Road, London, SW10 9NH, UK

Sena Jawad, s.jawad@imperial.ac.uk, Neonatal Data Analysis Unit, Imperial College London, Chelsea and Westminster Hospital campus, 369 Fulham Road, London, SW10 9NH, UK

Lucy Culshaw lucyculshaw@bliss.org.uk, Maya House, 134-138 Borough High Street, London, SE1 1LB

Jon Dorling jon.dorling@iwk.nshealth.ca Division of Neonatal-Perinatal Medicine, Faculty of Medicine, Dalhousie University, IWK Health Centre, 5850/5890 University Avenue, Halifax, Nova Scotia, B3K 6R8, Canada

Ursula Bowler ursula.bowler@npeu.ox.ac.uk National Perinatal Epidemiology Unit (Clinical Trials Unit), Nuffield Department of Population Health, University of Oxford, Old Road Campus, Headington, Oxford, OX3 7LF

Andrew King andy.king@npeu.ox.ac.uk National Perinatal Epidemiology Unit (Clinical Trials Unit), Nuffield Department of Population Health, University of Oxford, Old Road Campus, Headington, Oxford, OX3 7LF

For peer review only

Louise Linsell louise.linsell@npeu.ox.ac.uk National Perinatal Epidemiology Unit (Clinical Trials Unit), Nuffield Department of Population Health, University of Oxford, Old Road Campus, Headington, Oxford, OX3 7LF

Amanda Forster Research Nurse, amanda.forster2@nhs.net, Neonatal Unit, James Cook University Hospital, Marton Road, Middlesbrough, TS4 3BW

Jenny McLeish jenny.mcleish@npeu.ox.ac.uk National Perinatal Epidemiology Unit, Nuffield Department of Population Health, University of Oxford, Old Road Campus, Headington, Oxford, OX3 7LF

Mark Turner Mark.Turner@liverpool.ac.uk Women's and Children's Health, Institute of Translational Medicine, University of Liverpool, Liverpool, L69 3BX

Helen Robberts helenrobberts@hotmail.com c/o lucyculshaw@bliss.org.uk, Bliss, Maya House, 134-138 Borough High Street, London, SE1 1LB

Kayleigh Stanbury kayleigh.stanbury@npeu.ox.ac.uk National Perinatal Epidemiology Unit (Clinical Trials Unit), Nuffield Department of Population Health, University of Oxford, Old Road Campus, Headington, Oxford, OX3 7LF

Tjeerd-Pieter van Staa, tjeerd.vanstaa@manchester.ac.uk, Centre for Health Informatics, Division of Informatics, Imaging and Data Science, School of Health Sciences, Faculty of Biology, Medicine and Health, The University of Manchester, Manchester Academic Health Science Centre, Manchester, United Kingdom

Edmund Juszczak ed.juszczak@npeu.ox.ac.uk National Perinatal Epidemiology Unit (Clinical Trials Unit), Nuffield Department of Population Health, University of Oxford, Old Road Campus, Headington, Oxford, OX3 7LF

Corresponding author

For peer review only

Christopher Gale christopher.gale@imperial.ac.uk, Neonatal Medicine, Imperial College London, Chelsea and Westminster Hospital campus, 369 Fulham Road, London, SW10 9NH, UK

Keywords: randomised controlled trial, preterm infant, enteral feeding, transfusion, necrotising enterocolitis, electronic patient records, NNRD

For peer review only

For peer review only - http://bmjopen.bmj.com/site/about/guidelines.xhtml

ABSTRACT 333/350 words

Introduction

Necrotising enterocolitis (NEC) is a potentially devastating neonatal disease. A temporal association between red-cell transfusion and NEC is well described. Observational data suggest that withholding enteral feeds around red-cell transfusions may reduce the risk of NEC but this has not been tested in randomised trials; current UK practice varies. Prevention of NEC is a research priority but no appropriately powered trials have addressed this question. The use of a simplified opt-out consent model and embedding trial processes within existing electronic patient record (EPR) systems provide opportunities to increase trial efficiency and recruitment.

Methods and analysis

We will undertake a randomised, controlled, multi-centre, unblinded, pilot trial comparing two care pathways: continuing milk feeds (before, during and after red cell transfusions), and withholding milk feeds (for 4 hours before, during and for 4 hours after red cell transfusions), with infants randomly assigned with equal probability. We will use opt-out consent. A nested qualitative study will explore parent and health professional views. Infants will be eligible if born at $<30^{+0}$ gestational weeks^{+days}. Primary feasibility outcomes will be rate of recruitment, opt-out, retention, compliance, data completeness and data accuracy; clinical outcomes will include mortality and NEC. The trial will recruit in two neonatal networks in England for 9 months. Data collection will continue until all infants have reached 40^{+0} corrected gestational weeks or neonatal discharge. Participant identification and recruitment, randomisation and all trial data collection will be embedded within existing neonatal EPR systems

For peer review only

99 (BadgerNet and BadgerEPR); outcome data will be extracted from routinely recorded data held in the National Neonatal Research Database (NNRD).

Ethics and dissemination

This study holds Research Ethics Committee approval to use an opt-out approach to consent. Results will inform future EPR-embedded and data-enabled trials and will be disseminated through conferences, publications and parent-centred information.

ISRCTN registration: 62501859

For peer review only

For peer review only - http://bmjopen.bmj.com/site/about/guidelines.xhtml

Strengths and limitations

- NEC is a rare but potentially devastating neonatal disease, occurring predominantly in the most preterm infants. Neonatal trials to-date have not been adequately powered to detect realistic reductions in NEC.
- In this prospective, randomised pilot trial we will evaluate the feasibility of a data-enabled neonatal trial with processes embedded within an existing EPR system; accuracy and completeness of trial data will be validated at source.
- In this individually randomised, comparative effectiveness trial we will pilot opt-out consent and explore parent and health professional views of this approach in a nested qualitative study.
- We will evaluate the feasibility of EPR-embedded randomised comparative-effectiveness trials using a simplified opt-out consent for efficient, quicker and less resource burdensome neonatal trials at scale.

For peer review only

Background

Necrotising enterocolitis (NEC) is among the most potentially devastating neonatal diseases and has a mortality of up to 33%, the most severe form (requiring surgery or resulting in death) affects about 5% of infants born at less than 30 gestational weeks [1]; survivors are at high risk of long-term health [2] and developmental problems [3, 4]. Prevention of NEC has been identified as one of the most important research uncertainties in the field of preterm birth [5]. The pathogenesis of NEC is incompletely understood, however a temporal association between red cell transfusion and the subsequent development of the disease is well described [6, 7]. This "transfusion associated NEC" may be more severe [8] with higher mortality [9, 10]. The mechanism thought to underpin this relationship links milk feeds and packed red cell transfusion to NEC through altered mesenteric blood flow and intestinal barrier function; this is supported by animal (16) (17), and human studies (18, 19) (20, 21). Understanding the link between NEC and blood transfusion is of particular importance given that almost all very preterm babies will have a red cell transfusion and many will receive multiple transfusions (25).

Stopping milk feeds around the time of packed red cell transfusion is currently practised in some neonatal settings to reduce the risk of NEC, putatively by maintaining more physiological intestinal blood flow [11]. This practice has not however been tested in an adequately powered randomised trial, and there are physiological reasons why stopping milk feeds in preterm infants may lead to harm. Interrupting enteral feeding prolongs the time taken to reach full milk feeds, which is associated with invasive infection (23), and may paradoxically be associated with an increased risk of necrotising enterocolitis (24). One small, single-centre randomised pilot trial has assessed withholding enteral feeds around red cell transfusion but was

For peer review only - http://bmjopen.bmj.com/site/about/guidelines.xhtml

underpowered to detect a difference in NEC [12]. A systematic review of observational studies [13] identified seven historical control studies including 7,492 preterm infants; these studies were at high risk of bias including regression to the mean and ascertainment bias. Pooled results found an association between withholding feeds in the peri-transfusion period and a reduced risk of necrotising enterocolitis. The authors concluded that adequately powered randomised controlled trials are needed to confirm these findings.

There is considerable variation in current UK practice in relation to withholding enteral feeds during packed red cell transfusion in preterm infants: a 2011 survey of UK neonatal units (68% response rate) demonstrated that 35% of UK units routinely withheld enteral feeds during packed red cell transfusion [14].

If withholding enteral feeds around the time of packed red cell transfusion reduces the risk of NEC, then implementing this simple practice will reduce the mortality and long-term complications of NEC. Conversely, if the safety of continued feeding can be demonstrated, this will facilitate increased and consistent feeding with breastmilk, which has well described short and long-term benefits.

NEC is rare and occurs at a higher incidence in the most preterm infants and so trials targeting NEC need a large number of very preterm infants, who are themselves rare. As a result, no previous trial has been powered to look at NEC and there is no intervention to prevent NEC supported by high-quality randomised evidence. Methologies that have been proposed to improve efficiency and recruitment into randomised trials include the use of simplified opt-out approaches to consent [15], and embedding trial processes into existing Electronic Patient Record (EPR) systems [16].

The objectives of this pilot trial are:

For peer review only - http://bmjopen.bmj.com/site/about/guidelines.xhtml

For peer review only

1. To determine whether a large multi-centre trial addressing the following question is feasible: *Among preterm infants (Patient), does the practice of withholding enteral feeds around the time of blood transfusion (Intervention), compared with continued enteral feeding around the time of blood transfusion (Comparator), lead to a reduction in severe necrotising enterocolitis (Outcome)?*
2. To determine whether clinical trial processes (identifying participants, randomisation and data collection) can be successfully integrated into existing neonatal Electronic Patient Record (EPR) systems, and whether trial data can be extracted from routinely recorded clinical data held in the National Neonatal Research Database (NNRD).
3. To determine whether using a simplified opt-out consent process is feasible and acceptable to parents and health professionals.

METHODS

Design

The WHEAT trial is a randomised controlled, unblinded, multi-centre, pilot trial comparing two care pathways. The primary metrics of feasibility are recruitment, data completeness and data accuracy; clinical outcomes include mortality and NEC. Infants will be randomised with a 1:1 allocation ratio (using permuted blocks of variable size), stratified within neonatal unit by gestational age at birth and infant sex. Trial processes will be embedded within neonatal EPR systems and all outcome data will be extracted from data that are routinely recorded within the existing neonatal EPR systems (BadgerNet and BadgerEPR), and held in the National Neonatal Research Database (NNRD).

For peer review only - http://bmjopen.bmj.com/site/about/guidelines.xhtml

The trial will recruit infants from neonatal units within two neonatal networks in England: Northwest London Neonatal Network and Southern West Midlands Neonatal Operational Delivery Network. Recruitment will be for 9 months, with data collection continuing for a further 3 months, until all trial infants have finished follow-up at 40+0 corrected gestational weeks or neonatal discharge if sooner.

Eligibility criteria

Inclusion criteria:

- Preterm birth at less than 30^{+0} gestational weeks^{+days}

Exclusion criteria:

- Parent(s) opted out of trial participation
- Packed red cell transfusion with concurrent enteral feeds prior to enrolment. (Infants who have received a packed red cell transfusion while nil-by-mouth are eligible; buccal colostrum will not be counted as enteral feeding).
- Infants where enteral feeding is contraindicated in the first 7 days after birth (e.g. congenital abnormality).

Interventions

Both comparator pathways of care are standard in the UK; the WHEAT trial is a pilot comparative effectiveness trial. The two care pathways that will be compared are:

1. Withholding feeds around transfusion: all enteral feeds will be discontinued (the infant will be placed nil by mouth) for a period of 4 hours prior to packed red cell transfusion, during the packed red cell transfusion and until 4 hours post packed red cell transfusion. During this period (approximately 12 hours), hydration and

blood glucose will be maintained according to local practice, commonly by provision of parenteral nutrition or intravenous dextrose. Four hours after the red cell transfusion has finished, feeds will be restarted in the manner in which they were being received prior to the decision to transfuse. This duration of withholding feeds will follow the approach used in other trials [12] and observational studies [13], and identified to be the most acceptable in a survey of UK neonatal units.

2. Continuing feeds around transfusion: enteral feeds will continue to be given prior, during and after the packed red cell transfusion, in the manner in which they were being given prior to the decision to transfuse.

Infants will remain allocated to the same care pathway until 34+6 weeks+days gestational age.

In order to ensure that this pragmatic trial is as generalisable as possible to current practice, blood transfusions will be administered when clinically indicated according to local packed red cell transfusion guidelines. Data will be collected about pre-transfusion haemoglobin level for trial participants. Other concomitant care, including speed of increase of enteral feeds and choice of milk, for both the withholding feeds around transfusion pathway and the continuing feeds around transfusion pathway of care will be according to locally defined practice.

Outcomes

Feasibility outcomes:

1. Recruitment: proportion of infants <30 weeks of gestation admitted whose parents agree to trial involvement and the infant is randomised in the WHEAT trial

For peer review only

For peer review only - http://bmjopen.bmj.com/site/about/guidelines.xhtml

2. Retention: proportion of of recruited infants where outcome data are available up to the end of the follow-up period
3. Compliance: proportion of recruited infants who correctly received their allocated care pathway around all packed red cell transfusions between randomisation and 34^{+6} gestational $weeks^{+days}$
4. Data completeness: proportion of missing data for each data item reported as a baseline characteristic or an outcome
5. Data accuracy: proportion of cases where the following data items are correctly recorded when compared to source data (clinical notes or electronic patient record data)
    a. Severe NEC. All infants who had a diagnosis of non-severe NEC and a random sample of 25% of infants who did not have a diagnosis of NEC will have their source data verified to ensure that they do not meet the criteria for severe NEC
    b. Spontaneous intestinal perforation
    c. All-cause mortality
    d. Central line associated blood stream infection

Clinical outcomes:

All clinical outcomes will be assessed from randomisation to 40^{+0} weeks of gestation or neonatal unit discharge, whichever occurs first.

1. Severe NEC: histologically or surgically confirmed, or recorded in part 1 the death certificate. These infants will be identified as described in [17], which will include infants recorded as being transferred for surgery
2. Spontaneous intestinal perforation: histologically or surgically confirmed, or recorded in part 1 the death certificate.

For peer review only

3. All-cause mortality
4. Total duration of neonatal care in days: including all levels of care (intensive care, high dependency care, special care and ordinary care)
5. Duration of any parenteral nutrition in days
6. Number of days with a central venous line in situ
7. Number of central line associated blood stream infections defined according to National Neonatal Audit Programme (NNAP) 2017 definition [18]
8. Growth: change in birth weight and head circumference for gestational age standard deviation score

Sample size

There is no predefined sample size for this pilot trial. Recruitment (absolute numbers and the rate) will be a primary outcome for the pilot trial. The estimated recruitment target for the pilot trial is up to 250, based on predicted infant throughput at participating neonatal units and assuming 65–70% recruitment of eligible infants.

Data collection

Potential participants will be identified through the existing neonatal EPR systems that are widely used across England, Scotland and Wales; BadgerNet (a clinical summary system) or BadgerEPR (a complete electronic patient record system). Baseline data for all infants admitted to neonatal units in the UK are routinely entered into the EPR *admission summary* as part of normal clinical care. These data are updated in real-time and held securely on BadgerNet and BadgerEPR servers. In participating units, data entered electronically into the *admission summary* will be interrogated by the EPR platform in real time to identify and flag infants meeting the WHEAT trial inclusion criteria. When an infant in a participating unit meets the inclusion criteria, this will result

For peer review only - http://bmjopen.bmj.com/site/about/guidelines.xhtml

in an electronic reminder appearing on the EPR platform at the participating unit. This "flag" will inform the health professional that the infant is eligible for the WHEAT trial and link to the parent information leaflet. The EPR system will use data (neonatal unit, gestational age and sex) entered as part of the *admission summary* to stratify infants.

Baseline characteristics and clinical outcomes will be extracted from routinely recorded clinical data held in the NNRD. The NNRD holds data from all infants admitted to National Health Service (NHS) neonatal units in England, Scotland and Wales (approximately 90,000 infants annually). Contributing neonatal units are known as the UK Neonatal Collaborative (UKNC). Data are extracted from point-of-care neonatal electronic health records completed by health professionals during routine clinical care. A defined data extract, the Neonatal Dataset of approximately 450 data items [19], is transmitted quarterly to the Neonatal Data Analysis Unit at Imperial College London and Chelsea and Westminster NHS Foundation Trust where patient episodes across different hospitals are linked and data are cleaned (queries about discrepancies and implausible data configurations are fed back to health professionals and rectified) [20].

Randomisation

Infants will be randomly assigned to either pathway of care in a 1:1 allocation ratio as per a computer generated randomisation sequence using permuted blocks of various sizes with stratification as described below. The block sizes will not be disclosed to ensure allocation concealment.

Stratification will be by neonatal unit of enrolment and using the following categories:

1. Gestational age at birth

For peer review only

For peer review only - http://bmjopen.bmj.com/site/about/guidelines.xhtml

- <28^{+0} weeks^{+days}
- 28^{+0} to 29^{+6} weeks^{+days}

2. Infant sex

Infants that are part of a multiple birth set (twins, triplets or higher order multiples) will be randomised as a set to the same pathway of care following feedback from parent representatives, parent organisations including Bliss and TAMBA (Twins and Multiple Births Association) and research involving parents and adult ex-preterm twins [21].

Allocation concealment

Infants will be randomised using an online secure central randomisation system which will be embedded into the existing neonatal EPR systems (BadgerNet and BadgerEPR). Randomisation will occur within the EPR to ensure allocation concealment.

Blinding

The WHEAT trial will be unblinded as it is not possible to mask the different care pathways.

Statistical methods

The planned main WHEAT trial will be based on a superiority hypothesis; however, the pilot trial is not powered to detect any differences between the intervention arm (withholding feeds) and the comparator arm (continuing feeds).

Therefore, no formal statistical hypothesis testing will be conducted.

Continuous variables will be summarised using means and standard deviations unless their distributions are skewed, in which case medians, 25th quartiles, 75th quartiles and

For peer review only

For peer review only - http://bmjopen.bmj.com/site/about/guidelines.xhtml

the range (lowest and highest values) will be presented. Dichotomous variables will be presented as frequencies and percentages. In addition, 95% confidence intervals will be presented for the feasibility outcomes. The recruitment rate will be reported for both arms combined, and retention and compliance rates will be reported separately by treatment arm in addition to both arms combined.

Changes to the statistical analysis described in the original protocol

The following changes to the statistical analysis plan were made prior to completion of data collection:

- The pilot trial will not be performing any comparative analysis of outcomes between trial arms, or conducting any formal statistical hypothesis testing.
- The denominator for the recruitment rate will be infants <30 weeks of gestation admitted to recruiting sites; the planned denominator (infants that fulfill all of the eligibility criteria and whose parents have been approached) cannot be used as regulatory approval to use these data was not granted.
- The opt-out rate of parents whose infants are eligible for the trial will not be reported as regulatory approval to use these data was not granted.
- Data completeness will be reported for each individual data item and not the proportion of eligible infants for which trial items are complete.
- A random sample of 25% of infants who did not have a diagnosis of NEC recorded in the EPR system had their source data verified to ensure that they did not meet the criteria for severe NEC.

For peer review only - http://bmjopen.bmj.com/site/about/guidelines.xhtml

- All outcome events, including duration of hospital stay and growth scores, were be measured until neonatal unit discharge or 40^{+0} weeks of gestation, whichever occurs first.

Steering committee

An independent Trial Steering Committee (TSC) appointed by the study sponsor and approved by the funder (MRC) will oversee the project. The TSC will consist of an independent chair and at least two other independent members. The Chief Investigator and Clinical Trials Unit Director will also sit on the TSC.

Data monitoring

A Data Monitoring Committee (DMC) independent of the applicants and of the TSC will review the progress of the trial as agreed and provide advice on the conduct of the trial to the TSC and, via the TSC, to the Sponsor. The DMC will act according to its Charter, which will be agreed at its first meeting.

Adverse events

Due to the nature of the patient population, neonates in intensive care, a high incidence of adverse events is foreseeable during their routine care and treatment. Consequently, only those adverse events identified as serious (SAE) will be recorded for the trial. Unforeseen SAEs and the SAEs associated with the allocated pathway of care must be reported to the Clinical Trials Unit by a member of site staff within 24 hours of becoming aware of the event. Reporting of SAEs will not use existing EPR systems but will use telephone, fax and email systems.

Registration

For peer review only

For peer review only - http://bmjopen.bmj.com/site/about/guidelines.xhtml

This study is registered in ISRCTN (ISRCTN62501859).

Parent, patient and public involvement

The WHEAT pilot trial addresses one of the most important research uncertainties in preterm birth, as identified by over 500 parents, patients, health professionals and researchers [5]. The WHEAT trial has been developed in partnership with parents; protocol author HR is a parent with experience of preterm birth and protocol author LC represents Bliss, the charity for babies born premature or sick; both HR and Bliss have contributed to trial development from inception. Over 400 parents and patients have contributed to the selection of trial outcomes through the COIN project [22]. Parents and Bliss have been involved in developing the opt-out consent process, how this is communicated, in designing information leaflets and posters. The WHEAT trial has parent representatives on oversight committees to ensure that the trial

Ethics and dissemination

Research Ethics Committee approval was granted on 6th July 2018 by London - Bloomsbury Research Ethics Committee (18/LO/0900).

Because both the care pathways that are being compared are part of standard UK practice, WHEAT is using a simplified model of consent. This means that parents will have the WHEAT trial explained to them and will be asked to “opt out” if they do not want their infant to be randomised and enrolled in the trial. Parents will be approached shortly after their infant is admitted to the neonatal unit (in most cases within the first 24 hours). There is no upper time limit as to when trial discussions can take place. Parents will be able to opt out of the WHEAT trial at any point. Neonatal health professionals will be prompted within the EPR to explain WHEAT to parents of eligible

For peer review only - http://bmjopen.bmj.com/site/about/guidelines.xhtml

infants and to provide them with an information leaflet. If parents “opt out” this will be recorded in the EPR. If parents do not “opt out” and are happy for their infant to take part in WHEAT, randomisation will occur through the EPR. Enrolment of the infant and the allocation will be notified to the local team through the EPR. Because of the opt-out nature of WHEAT there will not be a signed consent form.

A qualitative exploration of the opt-out consent and recruitment process, and trial procedures will be conducted following the end of recruitment. Qualitative interviews will be undertaken with both parents that consented to the trial and health professionals from the recruiting sites.

Due to the common nature of packed red cell transfusion in the trial population (infants born at <30+0 gestational weeks+days), health professionals will explain the WHEAT trial and opt-out process shortly after birth (in most cases within the first 24 hours). A minority of infants will not receive a packed red cell transfusion during their neonatal unit stay. These will not be included in the main analysis population of clinical outcomes.

Results will be presented at national and international academic conferences and published in peer-reviewed scientific publications. Protocol author HR will work with the neonatal charity Bliss to produce parent-centred information for dissemination through social media, online and to be distributed on neonatal units.

Discussion

Preventing NEC is a recognised research priority in preterm birth [5], however there are no preventative interventions supported by high quality evidence. One key reason

For peer review only - http://bmjopen.bmj.com/site/about/guidelines.xhtml

is because NEC is a rare condition, therefore any trial seeking to detect a realistic reduction in NEC will require recruitment and randomisation of more preterm infants than ever previously achieved. For example, a trial seeking to detect a 25% relative risk reduction in NEC from a background rate of 6% [18] would need to randomise over 9,000 infants to have 90% power to detect such a difference with a two-sided 5% significance level. The largest previous individually randomised trial that included preterm infants was the INIS trial [23] which enrolled 3,493 infants. Undertaking neonatal trials on this scale will be challenging; for such large trials to be funded and sustainable, they will need to be more efficient, less burdensome and international in scope. There are successful examples of large simple trials in other specialties that can inform neonatal practice: the TASTE trial [24] demonstrated high efficiency and low burden by integrating trial processes within an existing data capture system, and the TRANSFUSE trial [25] demonstrated very high recruitment rates (>75%) through the use of opt-out models of consent. The WHEAT pilot trial will apply these approaches and measure their feasibility and acceptability in neonatal care. If these methodologies can be successfully applied, they will facilitiate efficient, large, simple trials suitable to address the many clinical uncertainties the plague neonatal care [26].

The WHEAT pilot trial will determine the feasibility of addressing an important clinical question regarding the optimal approach to feeding preterm infants around the time of red cell transfusions, in preparation for a future definitive trial. Currently there is insufficient evidence to recommend withholding or continuing milk feeds around red cell transfusion in preterm infants because available physiological and observational data are inconclusive.

Strength and limitations

For peer review only - http://bmjopen.bmj.com/site/about/guidelines.xhtml

The proposed trial has a number of strengths. The robustness of core NNRD data (birth weight, sex, length of stay and death) have been previously demonstrated for research purposes [1, 27, 28], this pilot trial will prospectively evaluate their accuracy and completeness for clinical trials. The trial will evaluate the feasibility of recruiting infants across two neonatal networks, including smaller neonatal units that do not traditionally recruit into neonatal randomised trials. Limitations include the unblinded nature of the trial and the use of a potentially subjective primary outcome, NEC. We endeavoured to mitigate against these through use of a previously validated, objective definition for NEC [1].

Conclusion

Neonatal trials to date have been unable to robustly evaluate strategies to prevent major preterm morbidities, such as optimal feeding around transfusion to prevent NEC, because of the large sample sizes required. This protocol describes a prospective, randomised controlled pilot trial to evaluate trial methodologies aiming to efficiently address such neonatal uncertainties.

Abbreviations

BSI blood stream infections

HQIP Healthcare Quality Improvement Partnership

NDAU Neonatal Data Analysis Unit

NEC necrotising enterocolitis

NNAP National Neonatal Audit Programme

For peer review only - http://bmjopen.bmj.com/site/about/guidelines.xhtml

NNRD National Neonatal Research Database

UK United Kingdom

UKNC United Kingdom Neonatal Collaborative

Consent for publication

Not applicable as the manuscript does not include any individual details.

Availability of data and material

The database to be used in this study is the NNRD which is managed at the Neonatal Data Analysis Unit directed by Professor Neena Modi; researchers, clinicians, managers, commissioners, and others are welcome and encouraged to utilise the NNRD. More details are available here: http://www.imperial.ac.uk/neonatal-data-analysis-unit/neonatal-data/utilising-the-nnrd/

Competing interests

The authors declare that they have no competing interests.

Funding

The trial is funded through a United Kingdom Medical Research Council (MRC) Clinician Scientist Fellowship awarded to CG.

Authors contributions

For peer review only

For peer review only - http://bmjopen.bmj.com/site/about/guidelines.xhtml

CG, NM, JD, AF, MT, HR, TPvS and CG conceived the study; all authors contributed to designing the study, developing the protocol for the study and this manuscript. All authors read and approved the final manuscript. HR is a parent of a preterm twins and LC is a representative of Bliss the charity for babies born premature or sick.

Acknowledgements

We are grateful to all the families that agreed to the inclusion of their baby's data in the NNRD, the health professionals who recorded data and the NDAU team, the members of the study steering group, and the members of the NU Neonatal Collaborative representing neonatal units that contribute data to the NNRD.

References

1. Battersby C, Longford NT, Mandalia S, Costeloe K, Modi N: **Incidence and enteral feed antecedents of severe neonatal necrotising enterocolitis across neonatal networks in England, 2012–13: a whole-population surveillance study**. *The Lancet Gastroenterology & Hepatology* 2017, **2**(1):43-51.
2. Duro D, Kalish LA, Johnston P, Jaksic T, McCarthy M, Martin C, Dunn JC, Brandt M, Nobuhara KK, Sylvester KG *et al*: **Risk factors for intestinal failure in infants with necrotizing enterocolitis: a Glaser Pediatric Research Network study**. *J Pediatr* 2010, **157**(2):203-208 e201.
3. Hintz SR, Kendrick DE, Stoll BJ, Vohr BR, Fanaroff AA, Donovan EF, Poole WK, Blakely ML, Wright L, Higgins R *et al*: **Neurodevelopmental and growth outcomes of extremely low birth weight infants after necrotizing enterocolitis**. *Pediatrics* 2005, **115**(3):696-703.
4. Rees CM, Pierro A, Eaton S: **Neurodevelopmental outcomes of neonates with medically and surgically treated necrotizing enterocolitis**. *Arch Dis Child Fetal Neonatal Ed* 2007, **92**(3):F193-198.
5. Duley L, Uhm S, Oliver S, Preterm Birth Priority Setting Partnership Steering G: **Top 15 UK research priorities for preterm birth**. *Lancet* 2014, **383**(9934):2041-2042.
6. Seges RA, Kenny A, Bird GW, Wingham J, Baals H, Stauffer UG: **Pediatric surgical patients with severe anaerobic infection: report of 16 T-antigen positive cases and possible hazards of blood transfusion**. *Journal of pediatric surgery* 1981, **16**(6):905-910.
7. Stritzke AI, Smyth J, Synnes A, Lee SK, Shah PS: **Transfusion-associated necrotising enterocolitis in neonates**. *Arch Dis Child Fetal Neonatal Ed* 2013, **98**(1):F10-14.
8. Blau J, Calo JM, Dozor D, Sutton M, Alpan G, La Gamma EF: **Transfusion-related acute gut injury: necrotizing enterocolitis in very low birth weight neonates after packed red blood cell transfusion**. *J Pediatr* 2011, **158**(3):403-409.
9. Mally P, Golombek SG, Mishra R, Nigam S, Mohandas K, Depalhma H, LaGamma EF: **Association of necrotizing enterocolitis with elective packed red blood cell transfusions in stable, growing, premature neonates**. *Am J Perinatol* 2006, **23**(8):451-458.

For peer review only - http://bmjopen.bmj.com/site/about/guidelines.xhtml

10. Cunningham KE, Okolo FC, Baker R, Mollen KP, Good M: **Red blood cell transfusion in premature infants leads to worse necrotizing enterocolitis outcomes**. *J Surg Res* 2017, **213**:158-165.

11. El-Dib M, Narang S, Lee E, Massaro AN, Aly H: **Red blood cell transfusion, feeding and necrotizing enterocolitis in preterm infants**. *J Perinatol* 2011, **31**(3):183-187.

12. Sahin S, Gozde Kanmaz Kutman H, Bozkurt O, Yavanoglu Atay F, Emre Canpolat F, Uras N, Suna Oguz S, Underwood MA: **Effect of withholding feeds on transfusion-related acute gut injury in preterm infants: a pilot randomized controlled trial**. *J Matern Fetal Neonatal Med* 2019:1-6.

13. Jasani B, Rao S, Patole S: **Withholding Feeds and Transfusion-Associated Necrotizing Enterocolitis in Preterm Infants: A Systematic Review**. *Adv Nutr* 2017, **8**(5):764-769.

14. Parige R, Turner C, Sundaram S, Power S: **Enteral feeding during packed red blood cell transfusion in English neonatal units**. *Arch Dis Child Fetal Neonatal Ed* 2013.

15. Gale C, Hyde MJ, Modi N, group Wtd: **Research ethics committee decision-making in relation to an efficient neonatal trial**. *Arch Dis Child Fetal Neonatal Ed* 2016.

16. van Staa TP, Dyson L, McCann G, Padmanabhan S, Belatri R, Goldacre B, Cassell J, Pirmohamed M, Torgerson D, Ronaldson S *et al*: **The opportunities and challenges of pragmatic point-of-care randomised trials using routinely collected electronic records: evaluations of two exemplar trials**. *Health Technol Assess* 2014, **18**(43):1-146.

17. Battersby C, Longford N, Costeloe K, Modi N, Group UKNCNES: **Development of a Gestational Age-Specific Case Definition for Neonatal Necrotizing Enterocolitis**. *JAMA Pediatr* 2017.

18. RCPCH: **National Neonatal Audit Programme (NNAP) 2018 annual report on 2017 data**. In. London: Royal College of Paediatrics and Child Health (RCPCH); 2018.

19. Digital N: **National Neonatal Data Set**. In. Edited by Dictionary ND, 3 edn. http://www.datadictionary.nhs.uk/web_site_content/navigation/national_neonatal_data_sets_menu.asp; 2016.

20. Spencer A, Modi N: **National neonatal data to support specialist care and improve infant outcomes**. *Archives of Disease in Childhood - Fetal and Neonatal Edition* 2012.

21. Bernardo J, Nowacki A, Martin R, Fanaroff JM, Hibbs AM: **Multiples and parents of multiples prefer same arm randomization of siblings in neonatal trials**. *J Perinatol* 2015, **35**(3):208-213.

22. Webbe J, Brunton G, Ali S, Duffy JMN, Modi N, Gale C: **Developing, implementing and disseminating a core outcome set for neonatal medicine**. *BMJ Paediatrics Open* 2017, **1**(e000048).

23. Group IC, Brocklehurst P, Farrell B, King A, Juszczak E, Darlow B, Haque K, Salt A, Stenson B, Tarnow-Mordi W: **Treatment of neonatal sepsis with intravenous immune globulin**. *N Engl J Med* 2011, **365**(13):1201-1211.

24. Frobert O, Lagerqvist B, Olivecrona GK, Omerovic E, Gudnason T, Maeng M, Aasa M, Angeras O, Calais F, Danielewicz M *et al*: **Thrombus aspiration during ST-segment elevation myocardial infarction**. *N Engl J Med* 2013, **369**(17):1587-1597.

25. Cooper DJ, McQuilten ZK, Nichol A, Ady B, Aubron C, Bailey M, Bellomo R, Gantner D, Irving DO, Kaukonen KM *et al*: **Age of Red Cells for Transfusion and Outcomes in Critically Ill Adults**. *N Engl J Med* 2017, **377**(19):1858-1867.

26. Willhelm C, Girisch W, Gottschling S, Graber S, Wahl H, Meyer S: **Systematic Cochrane reviews in neonatology: a critical appraisal**. *Pediatr Neonatol* 2013, **54**(4):261-266.

27. Gale C, Santhakumaran S, Nagarajan S, Statnikov Y, Modi N: **Impact of managed clinical networks on NHS specialist neonatal services in England: population based study**. *BMJ* 2012, **344**:e2105.

28. Battersby C, Statnikov Y, Santhakumaran S, Gray D, Modi N, Costeloe K, Collaborative UKN, Medicines for Neonates Investigator G: **The United Kingdom National Neonatal Research Database: A validation study**. *PLoS One* 2018, **13**(8):e0201815.

For peer review only - http://bmjopen.bmj.com/site/about/guidelines.xhtml

SPIRIT

STANDARD PROTOCOL ITEMS: RECOMMENDATIONS FOR INTERVENTIONAL TRIALS

SPIRIT 2013 Checklist: Recommended items to address in a clinical trial protocol and related documents*

Section/item	Item No	Description	Page
**Administrative information**			
Title	1	Descriptive title identifying the study design, population, interventions, and, if applicable, trial acronym	1
Trial registration	2a	Trial identifier and registry name. If not yet registered, name of intended registry	6, 19
	2b	All items from the World Health Organization Trial Registration Data Set	6, 19
Protocol version	3	Date and version identifier	NA
Funding	4	Sources and types of financial, material, and other support	23-24
Roles and responsibilities	5a	Names, affiliations, and roles of protocol contributors	1-3
	5b	Name and contact information for the trial sponsor	NA
	5c	Role of study sponsor and funders, if any, in study design; collection, management, analysis, and interpretation of data; writing of the report; and the decision to submit the report for publication, including whether they will have ultimate authority over any of these activities	23-24
	5d	Composition, roles, and responsibilities of the coordinating centre, steering committee, endpoint adjudication committee, data management team, and other individuals or groups overseeing the trial, if applicable (see Item 21a for data monitoring committee)	18
**Introduction**			
Background and rationale	6a	Description of research question and justification for undertaking the trial, including summary of relevant studies (published and unpublished) examining benefits and harms for each intervention	8-9
	6b	Explanation for choice of comparators	11-12

For peer review only

Objectives	7	Specific objectives or hypotheses	10
Trial design	8	Description of trial design including type of trial (eg, parallel group, crossover, factorial, single group), allocation ratio, and framework (eg, superiority, equivalence, noninferiority, exploratory)	10
**Methods: Participants, interventions, and outcomes**			
Study setting	9	Description of study settings (eg, community clinic, academic hospital) and list of countries where data will be collected. Reference to where list of study sites can be obtained	11
Eligibility criteria	10	Inclusion and exclusion criteria for participants. If applicable, eligibility criteria for study centres and individuals who will perform the interventions (eg, surgeons, psychotherapists)	11
Interventions	11a	Interventions for each group with sufficient detail to allow replication, including how and when they will be administered	11-12
	11b	Criteria for discontinuing or modifying allocated interventions for a given trial participant (eg, drug dose change in response to harms, participant request, or improving/worsening disease)	12
	11c	Strategies to improve adherence to intervention protocols, and any procedures for monitoring adherence (eg, drug tablet return, laboratory tests)	12
	11d	Relevant concomitant care and interventions that are permitted or prohibited during the trial	12
Outcomes	12	Primary, secondary, and other outcomes, including the specific measurement variable (eg, systolic blood pressure), analysis metric (eg, change from baseline, final value, time to event), method of aggregation (eg, median, proportion), and time point for each outcome. Explanation of the clinical relevance of chosen efficacy and harm outcomes is strongly recommended	12-13
Participant timeline	13	Time schedule of enrolment, interventions (including any run-ins and washouts), assessments, and visits for participants. A schematic diagram is highly recommended (see Figure)	12
Sample size	14	Estimated number of participants needed to achieve study objectives and how it was determined, including clinical and statistical assumptions supporting any sample size calculations	14
Recruitment	15	Strategies for achieving adequate participant enrolment to reach target sample size	14-15

Methods: Assignment of interventions (for controlled trials)

Allocation:

Sequence generation	16a	Method of generating the allocation sequence (eg, computer-generated random numbers), and list of any factors for stratification. To reduce predictability of a random sequence, details of any planned restriction (eg, blocking) should be provided in a separate document that is unavailable to those who enrol participants or assign interventions	16
Allocation concealment mechanism	16b	Mechanism of implementing the allocation sequence (eg, central telephone; sequentially numbered, opaque, sealed envelopes), describing any steps to conceal the sequence until interventions are assigned	15-16
Implementation	16c	Who will generate the allocation sequence, who will enrol participants, and who will assign participants to interventions	15-16
Blinding (masking)	17a	Who will be blinded after assignment to interventions (eg, trial participants, care providers, outcome assessors, data analysts), and how	16
	17b	If blinded, circumstances under which unblinding is permissible, and procedure for revealing a participant's allocated intervention during the trial	NA

Methods: Data collection, management, and analysis

Data collection methods	18a	Plans for assessment and collection of outcome, baseline, and other trial data, including any related processes to promote data quality (eg, duplicate measurements, training of assessors) and a description of study instruments (eg, questionnaires, laboratory tests) along with their reliability and validity, if known. Reference to where data collection forms can be found, if not in the protocol	14-15
	18b	Plans to promote participant retention and complete follow-up, including list of any outcome data to be collected for participants who discontinue or deviate from intervention protocols	14-15
Data management	19	Plans for data entry, coding, security, and storage, including any related processes to promote data quality (eg, double data entry; range checks for data values). Reference to where details of data management procedures can be found, if not in the protocol	14-15
Statistical methods	20a	Statistical methods for analysing primary and secondary outcomes. Reference to where other details of the statistical analysis plan can be found, if not in the protocol	16-18

	20b	Methods for any additional analyses (eg, subgroup and adjusted analyses)	16-18
	20c	Definition of analysis population relating to protocol non-adherence (eg, as randomised analysis), and any statistical methods to handle missing data (eg, multiple imputation)	16-18
**Methods: Monitoring**			
Data monitoring	21a	Composition of data monitoring committee (DMC); summary of its role and reporting structure; statement of whether it is independent from the sponsor and competing interests; and reference to where further details about its charter can be found, if not in the protocol. Alternatively, an explanation of why a DMC is not needed	18
	21b	Description of any interim analyses and stopping guidelines, including who will have access to these interim results and make the final decision to terminate the trial	18
Harms	22	Plans for collecting, assessing, reporting, and managing solicited and spontaneously reported adverse events and other unintended effects of trial interventions or trial conduct	18-19
Auditing	23	Frequency and procedures for auditing trial conduct, if any, and whether the process will be independent from investigators and the sponsor	15
**Ethics and dissemination**			
Research ethics approval	24	Plans for seeking research ethics committee/institutional review board (REC/IRB) approval	19
Protocol amendments	25	Plans for communicating important protocol modifications (eg, changes to eligibility criteria, outcomes, analyses) to relevant parties (eg, investigators, REC/IRBs, trial participants, trial registries, journals, regulators)	17-18
Consent or assent	26a	Who will obtain informed consent or assent from potential trial participants or authorised surrogates, and how (see Item 32)	19
	26b	Additional consent provisions for collection and use of participant data and biological specimens in ancillary studies, if applicable	19
Confidentiality	27	How personal information about potential and enrolled participants will be collected, shared, and maintained in order to protect confidentiality before, during, and after the trial	14-15
Declaration of interests	28	Financial and other competing interests for principal investigators for the overall trial and each study site	23

For peer review only - http://bmjopen.bmj.com/site/about/guidelines.xhtml

Access to data	29	Statement of who will have access to the final trial dataset, and disclosure of contractual agreements that limit such access for investigators	23
Ancillary and post-trial care	30	Provisions, if any, for ancillary and post-trial care, and for compensation to those who suffer harm from trial participation	NA
Dissemination policy	31a	Plans for investigators and sponsor to communicate trial results to participants, healthcare professionals, the public, and other relevant groups (eg, via publication, reporting in results databases, or other data sharing arrangements), including any publication restrictions	19-20
	31b	Authorship eligibility guidelines and any intended use of professional writers	24
	31c	Plans, if any, for granting public access to the full protocol, participant-level dataset, and statistical code	NA
**Appendices**			
Informed consent materials	32	Model consent form and other related documentation given to participants and authorised surrogates	NA
Biological specimens	33	Plans for collection, laboratory evaluation, and storage of biological specimens for genetic or molecular analysis in the current trial and for future use in ancillary studies, if applicable	NA

*It is strongly recommended that this checklist be read in conjunction with the SPIRIT 2013 Explanation & Elaboration for important clarification on the items. Amendments to the protocol should be tracked and dated. The SPIRIT checklist is copyrighted by the SPIRIT Group under the Creative Commons "Attribution-NonCommercial-NoDerivs 3.0 Unported" license.

For peer review only

BMJ Open

Study Protocol: The WHEAT pilot trial - WithHolding Enteral feeds Around packed red cell Transfusion to prevent necrotising enterocolitis in preterm neonates: a multi-centre, electronic patient record (EPR), randomised controlled point-of-care pilot trial

Journal:	*BMJ Open*
Manuscript ID	bmjopen-2019-033543.R1
Article Type:	Protocol
Date Submitted by the Author:	03-Sep-2019
Complete List of Authors:	Gale, Chris; Imperial College London, Neonatal Medicine, School of Public Health, Chelsea and Westminster campus<br>Modi, Neena; Imperial College London, Neonatal Medicine, School of Public Health, Chelsea and Westminster campus<br>Jawad, Sena; Imperial College London, Neonatal Data Analysis Unit, School of Public Health, Chelsea and Westminster campus<br>Culshaw, Lucy; Bliss – The National Charity for the Newborn<br>Dorling, Jon; Dalhousie University, IWK Health Centre, Division of Neonatal-Perinatal Medicine, Faculty of Medicine<br>Bowler, Ursula; National Perinatal Epidemiology Unit, Clinical Trials Unit, Nuffield Department of Population Health, University of Oxford, Old Road Campus<br>Forster, Amanda; James Cook University Hospital, Neonatal Uni<br>King, Andy; National Perinatal Epidemiology Unit, Clinical Trials Unit, Nuffield Department of Population Health, University of Oxford, Old Road Campus<br>McLeish, Jenny; National Perinatal Epidemiology Unit, Nuffield Department of Population Health, University of Oxford, Old Road Campus<br>Linsell, Louise; National Perinatal Epidemiology Unit, Clinical Trials Unit, Nuffield Department of Population Health, University of Oxford, Old Road Campus<br>Turner, Mark; University of Liverpool, Women's and Children's Health, Institute of Translational Medicine<br>Robberts, Helen; Bliss – The National Charity for the Newborn, Parent of preterm twins<br>Stanbury, Kayleigh; National Perinatal Epidemiology Unit, Clinical Trials Unit, Nuffield Department of Population Health, University of Oxford, Old Road Campus<br>van Staa, Tjeerd; The University of Manchester, Centre for Health Informatics, Division of Informatics, Imaging and Data Science, School of Health Sciences, Faculty of Biology, Medicine and Health<br>Juszczak, Ed; National Perinatal Epidemiology Unit, Clinical Trials Unit, Nuffield Department of Population Health, University of Oxford, Old Road Campus
**Primary Subject Heading**:	Paediatrics

Secondary Subject Heading:	Intensive care, Haematology (incl blood transfusion), Nutrition and metabolism
Keywords:	randomised controlled trial, preterm infant, Blood bank & transfusion medicine < HAEMATOLOGY, NEONATOLOGY, NNRD, electronic patient records

Title page

Study Protocol: The WHEAT pilot trial - WithHolding Enteral feeds Around packed red cell Transfusion to prevent necrotising enterocolitis in preterm neonates: a multi-centre, electronic patient record (EPR), randomised controlled point-of-care pilot trial

Gale C[1], Modi N[1], Jawad S[1], Culshaw L[2], Dorling J[3], Bowler U[4], Forster A[5], King A[4], McLeish J[4], Linsell L[4], Turner M[6], Robberts H[7], Stanbury K[4], van Staa T[8], Juszczak E[4]

Institutions

[1] Neonatal Data Analysis Unit, Imperial College London, UK

[2] Bliss – The National Charity for the Newborn, Chapter House, 18-20 Crucifix Lane, London, SE1 3JW

[3] Division of Neonatal-Perinatal Medicine, IWK Health Centre, Dalhousie University, Halifax, NS, Canada B3H 4R2.

[4] National Perinatal Epidemiology Unit, Nuffield Department of Population Health, University of Oxford

[5] Research Nurse, Neonatal Unit, James Cook University Hospital, Marton Road, Middlesbrough, TS4 3BW

[6] Professor of Neonatology and Research Delivery, Women's and Children's Health, Institute of Translational Medicine, University of Liverpool, Liverpool, L69 3BX

[7] Parent of preterm twins, c/o Bliss – The National Charity for the Newborn, Chapter House, 18-20 Crucifix Lane, London, SE1 3JW

For peer review only - http://bmjopen.bmj.com/site/about/guidelines.xhtml

[8] Professor in Health e-Research, Centre for Health Informatics, Division of Informatics, Imaging and Data Science, School of Health Sciences, Faculty of Biology, Medicine and Health, The University of Manchester, Manchester Academic Health Science Centre, Manchester, United Kingdom

Authors

Neena Modi n.modi@imperial.ac.uk, Neonatal Data Analysis Unit, Imperial College London, Chelsea and Westminster Hospital campus, 369 Fulham Road, London, SW10 9NH, UK

Sena Jawad, s.jawad@imperial.ac.uk, Neonatal Data Analysis Unit, Imperial College London, Chelsea and Westminster Hospital campus, 369 Fulham Road, London, SW10 9NH, UK

Lucy Culshaw lucyculshaw@bliss.org.uk, Maya House, 134-138 Borough High Street, London, SE1 1LB

Jon Dorling jon.dorling@iwk.nshealth.ca Division of Neonatal-Perinatal Medicine, Faculty of Medicine, Dalhousie University, IWK Health Centre, 5850/5890 University Avenue, Halifax, Nova Scotia, B3K 6R8, Canada

Ursula Bowler ursula.bowler@npeu.ox.ac.uk National Perinatal Epidemiology Unit (Clinical Trials Unit), Nuffield Department of Population Health, University of Oxford, Old Road Campus, Headington, Oxford, OX3 7LF

Andrew King andy.king@npeu.ox.ac.uk National Perinatal Epidemiology Unit (Clinical Trials Unit), Nuffield Department of Population Health, University of Oxford, Old Road Campus, Headington, Oxford, OX3 7LF

For peer review only - http://bmjopen.bmj.com/site/about/guidelines.xhtml

Louise Linsell louise.linsell@npeu.ox.ac.uk National Perinatal Epidemiology Unit (Clinical Trials Unit), Nuffield Department of Population Health, University of Oxford, Old Road Campus, Headington, Oxford, OX3 7LF

Amanda Forster Research Nurse, amanda.forster2@nhs.net, Neonatal Unit, James Cook University Hospital, Marton Road, Middlesbrough, TS4 3BW

Jenny McLeish jenny.mcleish@npeu.ox.ac.uk National Perinatal Epidemiology Unit, Nuffield Department of Population Health, University of Oxford, Old Road Campus, Headington, Oxford, OX3 7LF

Mark Turner Mark.Turner@liverpool.ac.uk Women's and Children's Health, Institute of Translational Medicine, University of Liverpool, Liverpool, L69 3BX

Helen Robberts helenrobberts@hotmail.com c/o lucyculshaw@bliss.org.uk, Bliss, Maya House, 134-138 Borough High Street, London, SE1 1LB

Kayleigh Stanbury kayleigh.stanbury@npeu.ox.ac.uk National Perinatal Epidemiology Unit (Clinical Trials Unit), Nuffield Department of Population Health, University of Oxford, Old Road Campus, Headington, Oxford, OX3 7LF

Tjeerd-Pieter van Staa, tjeerd.vanstaa@manchester.ac.uk, Centre for Health Informatics, Division of Informatics, Imaging and Data Science, School of Health Sciences, Faculty of Biology, Medicine and Health, The University of Manchester, Manchester Academic Health Science Centre, Manchester, United Kingdom

Edmund Juszczak ed.juszczak@npeu.ox.ac.uk National Perinatal Epidemiology Unit (Clinical Trials Unit), Nuffield Department of Population Health, University of Oxford, Old Road Campus, Headington, Oxford, OX3 7LF

Corresponding author

For peer review only

Christopher Gale christopher.gale@imperial.ac.uk, Neonatal Medicine, Imperial College London, Chelsea and Westminster Hospital campus, 369 Fulham Road, London, SW10 9NH, UK

Keywords: randomised controlled trial, preterm infant, enteral feeding, transfusion, necrotising enterocolitis, electronic patient records, NNRD

For peer review only

ABSTRACT 333/350 words

Introduction

Necrotising enterocolitis (NEC) is a potentially devastating neonatal disease. A temporal association between red-cell transfusion and NEC is well described. Observational data suggest that withholding enteral feeds around red-cell transfusions may reduce the risk of NEC but this has not been tested in randomised trials; current UK practice varies. Prevention of NEC is a research priority but no appropriately powered trials have addressed this question. The use of a simplified opt-out consent model and embedding trial processes within existing electronic patient record (EPR) systems provide opportunities to increase trial efficiency and recruitment.

Methods and analysis

We will undertake a randomised, controlled, multi-centre, unblinded, pilot trial comparing two care pathways: continuing milk feeds (before, during and after red cell transfusions), and withholding milk feeds (for 4 hours before, during and for 4 hours after red cell transfusions), with infants randomly assigned with equal probability. We will use opt-out consent. A nested qualitative study will explore parent and health professional views. Infants will be eligible if born at <30^{+0} gestational weeks^{+days}. Primary feasibility outcomes will be rate of recruitment, opt-out, retention, compliance, data completeness and data accuracy; clinical outcomes will include mortality and NEC. The trial will recruit in two neonatal networks in England for 9 months. Data collection will continue until all infants have reached 40^{+0} corrected gestational weeks or neonatal discharge. Participant identification and recruitment, randomisation and all trial data collection will be embedded within existing neonatal EPR systems

For peer review only - http://bmjopen.bmj.com/site/about/guidelines.xhtml

(BadgerNet and BadgerEPR); outcome data will be extracted from routinely recorded data held in the National Neonatal Research Database (NNRD).

Ethics and dissemination

This study holds Research Ethics Committee approval to use an opt-out approach to consent. Results will inform future EPR-embedded and data-enabled trials and will be disseminated through conferences, publications and parent-centred information.

ISRCTN registration: 62501859

For peer review only

For peer review only - http://bmjopen.bmj.com/site/about/guidelines.xhtml

Strengths and limitations

- NEC is a rare but potentially devastating neonatal disease, occurring predominantly in the most preterm infants. Neonatal trials to-date have not been adequately powered to detect realistic reductions in NEC.
- In this prospective, randomised pilot trial we will evaluate the feasibility of a data-enabled neonatal trial with processes embedded within an existing EPR system; accuracy and completeness of trial data will be validated at source.
- In this individually randomised, comparative effectiveness trial we will pilot opt-out consent and explore parent and health professional views of this approach in a nested qualitative study.
- We will evaluate the feasibility of EPR-embedded randomised comparative-effectiveness trials using a simplified opt-out consent for efficient, quicker and less resource burdensome neonatal trials at scale.

For peer review only - http://bmjopen.bmj.com/site/about/guidelines.xhtml

Background

Necrotising enterocolitis (NEC) is among the most potentially devastating neonatal diseases and has a mortality of up to 33%, the most severe form (requiring surgery or resulting in death) affects about 5% of infants born at less than 30 gestational weeks [1]; survivors are at high risk of long-term health [2] and developmental problems [3, 4]. Prevention of NEC has been identified as one of the most important research uncertainties in the field of preterm birth [5]. The pathogenesis of NEC is incompletely understood, however a temporal association between red cell transfusion and the subsequent development of the disease is well described [6, 7]. This "transfusion associated NEC" may be more severe [8] with higher mortality [9, 10]. The mechanism thought to underpin this relationship links milk feeds and packed red cell transfusion to NEC through altered mesenteric blood flow and intestinal barrier function; this is supported by animal (16) (17), and human studies (18, 19) (20, 21). Understanding the link between NEC and blood transfusion is of particular importance given that almost all very preterm babies will have a red cell transfusion and many will receive multiple transfusions (25).

Stopping milk feeds around the time of packed red cell transfusion is currently practised in some neonatal settings to reduce the risk of NEC, putatively by maintaining more physiological intestinal blood flow [11]. This practice has not however been tested in an adequately powered randomised trial, and there are physiological reasons why stopping milk feeds in preterm infants may lead to harm. Interrupting enteral feeding prolongs the time taken to reach full milk feeds, which is associated with invasive infection (23), and may paradoxically be associated with an increased risk of necrotising enterocolitis (24). One small, single-centre randomised pilot trial has assessed withholding enteral feeds around red cell transfusion but was

For peer review only - http://bmjopen.bmj.com/site/about/guidelines.xhtml

underpowered to detect a difference in NEC [12]. A systematic review of observational studies [13] identified seven historical control studies including 7,492 preterm infants; these studies were at high risk of bias including regression to the mean and ascertainment bias. Pooled results found an association between withholding feeds in the peri-transfusion period and a reduced risk of necrotising enterocolitis. The authors concluded that adequately powered randomised controlled trials are needed to confirm these findings.

There is considerable variation in current UK practice in relation to withholding enteral feeds during packed red cell transfusion in preterm infants: a 2011 survey of UK neonatal units (68% response rate) demonstrated that 35% of UK units routinely withheld enteral feeds during packed red cell transfusion [14].

If withholding enteral feeds around the time of packed red cell transfusion reduces the risk of NEC, then implementing this simple practice will reduce the mortality and long-term complications of NEC. Conversely, if the safety of continued feeding can be demonstrated, this will facilitate increased and consistent feeding with breastmilk, which has well described short and long-term benefits.

NEC is rare and occurs at a higher incidence in the most preterm infants and so trials targeting NEC need a large number of very preterm infants, who are themselves rare. As a result, no previous trial has been powered to look at NEC and there is no intervention to prevent NEC supported by high-quality randomised evidence. Methologies that have been proposed to improve efficiency and recruitment into randomised trials include the use of simplified opt-out approaches to consent [15], and embedding trial processes into existing Electronic Patient Record (EPR) systems [16].

The objectives of this pilot trial are:

For peer review only - http://bmjopen.bmj.com/site/about/guidelines.xhtml

1. To determine whether a large multi-centre trial addressing the following question is feasible: *Among preterm infants (Patient), does the practice of withholding enteral feeds around the time of blood transfusion (Intervention), compared with continued enteral feeding around the time of blood transfusion (Comparator), lead to a reduction in severe necrotising enterocolitis (Outcome)?*
2. To determine whether clinical trial processes (identifying participants, randomisation and data collection) can be successfully integrated into existing neonatal Electronic Patient Record (EPR) systems, and whether trial data can be extracted from routinely recorded clinical data held in the National Neonatal Research Database (NNRD).
3. To determine whether using a simplified opt-out consent process is feasible and acceptable to parents and health professionals.

For peer review only

METHODS

Design

The WHEAT trial is a randomised controlled, unblinded, multi-centre, pilot trial comparing two care pathways. The primary metrics of feasibility are recruitment, data completeness and data accuracy; clinical outcomes include mortality and NEC. Infants will be randomised with a 1:1 allocation ratio (using permuted blocks of variable size), stratified within neonatal unit by gestational age at birth and infant sex. Trial processes will be embedded within neonatal EPR systems and all outcome data will be extracted from data that are routinely recorded within the existing neonatal EPR systems (BadgerNet and BadgerEPR), and held in the National Neonatal Research Database (NNRD).

For peer review only - http://bmjopen.bmj.com/site/about/guidelines.xhtml

The trial will recruit infants from neonatal units within two neonatal networks in England: Northwest London Neonatal Network and Southern West Midlands Neonatal Operational Delivery Network. Recruitment will be for 9 months (15th October 2018 – 30th June 2019), with data collection continuing for a further 3 months, until all trial infants have finished follow-up at 40+0 corrected gestational weeks or neonatal discharge if sooner.

Eligibility criteria

Inclusion criteria:

- Preterm birth at less than 30^{+0} gestational $weeks^{+days}$

Exclusion criteria:

- Parent(s) opted out of trial participation
- Packed red cell transfusion with concurrent enteral feeds prior to enrolment. (Infants who have received a packed red cell transfusion while nil-by-mouth are eligible; buccal colostrum will not be counted as enteral feeding).
- Infants where enteral feeding is contraindicated in the first 7 days after birth (e.g. congenital abnormality).

Interventions

Both comparator pathways of care are standard in the UK; the WHEAT trial is a pilot comparative effectiveness trial. The two care pathways that will be compared are:

1. Withholding feeds around transfusion: all enteral feeds will be discontinued (the infant will be placed nil by mouth) for a period of 4 hours prior to packed red cell transfusion, during the packed red cell transfusion and until 4 hours post packed

For peer review only - http://bmjopen.bmj.com/site/about/guidelines.xhtml

red cell transfusion. During this period (approximately 12 hours), hydration and blood glucose will be maintained according to local practice, commonly by provision of parenteral nutrition or intravenous dextrose. Four hours after the red cell transfusion has finished, feeds will be restarted in the manner in which they were being received prior to the decision to transfuse. This duration of withholding feeds will follow the approach used in other trials [12] and observational studies [13], and identified to be the most acceptable in a survey of UK neonatal units.

2. Continuing feeds around transfusion: enteral feeds will continue to be given prior, during and after the packed red cell transfusion, in the manner in which they were being given prior to the decision to transfuse.

Infants will remain allocated to the same care pathway until 34+6 weeks+days gestational age.

In order to ensure that this pragmatic trial is as generalisable as possible to current practice, blood transfusions will be administered when clinically indicated according to local packed red cell transfusion guidelines. Data will be collected about pre-transfusion haemoglobin level for trial participants. Other concomitant care, including speed of increase of enteral feeds and choice of milk, for both the withholding feeds around transfusion pathway and the continuing feeds around transfusion pathway of care will be according to locally defined practice.

Outcomes

Feasibility outcomes:

For peer review only - http://bmjopen.bmj.com/site/about/guidelines.xhtml

1. Recruitment: proportion of infants <30 weeks of gestation admitted whose parents agree to trial involvement and the infant is randomised in the WHEAT trial
2. Retention: proportion of of recruited infants where outcome data are available up to the end of the follow-up period
3. Compliance: proportion of recruited infants who correctly received their allocated care pathway around all packed red cell transfusions between randomisation and 34^{+6} gestational weeks^{+days}
4. Data completeness: proportion of missing data for each data item reported as a baseline characteristic or an outcome
5. Data accuracy: proportion of cases where the following data items are correctly recorded when compared to source data (clinical notes or electronic patient record data)
    a. Severe NEC: All infants who had a diagnosis of non-severe NEC and a random sample of 25% of infants who did not have a diagnosis of NEC will have their source data verified to ensure that they do not meet the criteria for severe NEC; 25% was selected for pragmatic reasons
    b. Spontaneous intestinal perforation
    c. All-cause mortality
    d. Central line associated blood stream infection

Clinical outcomes:

All clinical outcomes will be assessed from randomisation to 40^{+0} weeks of gestation or neonatal unit discharge, whichever occurs first.

For peer review only

For peer review only - http://bmjopen.bmj.com/site/about/guidelines.xhtml

1. Severe NEC: histologically or surgically confirmed, or recorded in part 1 the death certificate. These infants will be identified as described in [17], which will include infants recorded as being transferred for surgery
2. Spontaneous intestinal perforation: histologically or surgically confirmed, or recorded in part 1 the death certificate.
3. All-cause mortality
4. Total duration of neonatal care in days: including all levels of care (intensive care, high dependency care, special care and ordinary care)
5. Duration of any parenteral nutrition in days
6. Number of days with a central venous line in situ
7. Number of central line associated blood stream infections defined according to National Neonatal Audit Programme (NNAP) 2017 definition [18]
8. Growth: change in birth weight and head circumference for gestational age standard deviation score

Sample size

There is no predefined sample size for this pilot trial. Recruitment (absolute numbers and the rate) will be a primary outcome for the pilot trial. The estimated recruitment target for the pilot trial is up to 250, based on predicted infant throughput at participating neonatal units and assuming 65–70% recruitment of eligible infants.

Data collection

Potential participants will be identified through the existing neonatal EPR systems that are widely used across England, Scotland and Wales; BadgerNet (a clinical summary system) or BadgerEPR (a complete electronic patient record system). Baseline data for all infants admitted to neonatal units in the UK are routinely entered into the EPR

For peer review only - http://bmjopen.bmj.com/site/about/guidelines.xhtml

admission summary as part of normal clinical care. These data are updated in real-time and held securely on BadgerNet and BadgerEPR servers. In participating units, data entered electronically into the *admission summary* will be interrogated by the EPR platform in real time to identify and flag infants meeting the WHEAT trial inclusion criteria. When an infant in a participating unit meets the inclusion criteria, this will result in an electronic reminder appearing on the EPR platform at the participating unit. This "flag" will inform the health professional that the infant is eligible for the WHEAT trial and link to the parent information leaflet. The EPR system will use data (neonatal unit, gestational age and sex) entered as part of the *admission summary* to stratify infants.

Baseline characteristics and clinical outcomes will be extracted from routinely recorded clinical data held in the NNRD. The NNRD holds data from all infants admitted to National Health Service (NHS) neonatal units in England, Scotland and Wales (approximately 90,000 infants annually). Contributing neonatal units are known as the UK Neonatal Collaborative (UKNC). Data are extracted from point-of-care neonatal electronic health records completed by health professionals during routine clinical care. A defined data extract, the Neonatal Dataset of approximately 450 data items [19], is transmitted quarterly to the Neonatal Data Analysis Unit at Imperial College London and Chelsea and Westminster NHS Foundation Trust where patient episodes across different hospitals are linked and data are cleaned (queries about discrepancies and implausible data configurations are fed back to health professionals and rectified) [20].

Randomisation

Infants will be randomly assigned to either pathway of care in a 1:1 allocation ratio as per a computer generated randomisation sequence using permuted blocks of various

For peer review only - http://bmjopen.bmj.com/site/about/guidelines.xhtml

sizes with stratification as described below. The block sizes will not be disclosed to ensure allocation concealment.

Stratification will be by neonatal unit of enrolment and using the following categories:

1. Gestational age at birth
    - $<28^{+0}$ weeks^{+days}
    - 28^{+0} to 29^{+6} weeks^{+days}
2. Infant sex

Infants that are part of a multiple birth set (twins, triplets or higher order multiples) will be randomised as a set to the same pathway of care following feedback from parent representatives, parent organisations including Bliss and TAMBA (Twins and Multiple Births Association) and research involving parents and adult ex-preterm twins [21].

Allocation concealment

Infants will be randomised using an online secure central randomisation system which will be embedded into the existing neonatal EPR systems (BadgerNet and BadgerEPR). Randomisation will occur within the EPR to ensure allocation concealment.

Blinding

The WHEAT trial will be unblinded as it is not possible to mask the different care pathways.

Statistical methods

For peer review only

The planned main WHEAT trial will be based on a superiority hypothesis; however, the pilot trial is not powered to detect any differences between the intervention arm (withholding feeds) and the comparator arm (continuing feeds).

Therefore, no formal statistical hypothesis testing will be conducted.

Continuous variables will be summarised using means and standard deviations unless their distributions are skewed, in which case medians, 25th quartiles, 75th quartiles and the range (lowest and highest values) will be presented. Dichotomous variables will be presented as frequencies and percentages. In addition, 95% confidence intervals will be presented for the feasibility outcomes. The recruitment rate will be reported for both arms combined, and retention and compliance rates will be reported separately by treatment arm in addition to both arms combined.

Changes to the statistical analysis described in the original protocol

The original protocol is available as supplementary data. The following changes to the statistical analysis plan were made prior to completion of data collection:

- The pilot trial will not be performing any comparative analysis of outcomes between trial arms, or conducting any formal statistical hypothesis testing.
- The denominator for the recruitment rate will be infants <30 weeks of gestation admitted to recruiting sites; the planned denominator (infants that fulfill all of the eligibility criteria and whose parents have been approached) cannot be used as regulatory approval to use these data was not granted.
- The opt-out rate of parents whose infants are eligible for the trial will not be reported as regulatory approval to use these data was not granted.

For peer review only - http://bmjopen.bmj.com/site/about/guidelines.xhtml

- Data completeness will be reported for each individual data item and not the proportion of eligible infants for which trial items are complete.
- A random sample of 25% of infants who did not have a diagnosis of NEC recorded in the EPR system had their source data verified to ensure that they did not meet the criteria for severe NEC.
- All outcome events, including duration of hospital stay and growth scores, were be measured until neonatal unit discharge or 40^{+0} weeks of gestation, whichever occurs first.

Steering committee

An independent Trial Steering Committee (TSC) appointed by the study sponsor and approved by the funder (MRC) will oversee the project. The TSC will consist of an independent chair and at least two other independent members. The Chief Investigator and Clinical Trials Unit Director will also sit on the TSC.

Data monitoring

A Data Monitoring Committee (DMC) independent of the applicants and of the TSC will review the progress of the trial as agreed and provide advice on the conduct of the trial to the TSC and, via the TSC, to the Sponsor. The DMC will act according to its Charter, which will be agreed at its first meeting.

Adverse events

Due to the nature of the patient population, neonates in intensive care, a high incidence of adverse events is foreseeable during their routine care and treatment. Consequently, only those adverse events identified as serious (SAE) will be recorded

For peer review only - http://bmjopen.bmj.com/site/about/guidelines.xhtml

for the trial. Unforeseen SAEs and the SAEs associated with the allocated pathway of care must be reported to the Clinical Trials Unit by a member of site staff within 24 hours of becoming aware of the event. Reporting of SAEs will not use existing EPR systems but will use telephone, fax and email systems.

Registration

This study is registered in ISRCTN (ISRCTN62501859).

Parent, patient and public involvement

The WHEAT pilot trial addresses one of the most important research uncertainties in preterm birth, as identified by over 500 parents, patients, health professionals and researchers [5]. The WHEAT trial has been developed in partnership with parents; protocol author HR is a parent with experience of preterm birth and protocol author LC represents Bliss, the charity for babies born premature or sick; both HR and Bliss have contributed to trial development from inception. Over 400 parents and patients have contributed to the selection of trial outcomes through the COIN project [22]. Parents and Bliss have been involved in developing the opt-out consent process, how this is communicated, in designing information leaflets and posters. The WHEAT trial has parent representatives on oversight committees to ensure that the trial

Ethics and dissemination

Research Ethics Committee approval was granted on 6th July 2018 by London - Bloomsbury Research Ethics Committee (18/LO/0900).

Because both the care pathways that are being compared are part of standard UK practice, WHEAT is using a simplified model of consent. This means that parents will have the WHEAT trial explained to them and will be asked to “opt out” if they do not

For peer review only - http://bmjopen.bmj.com/site/about/guidelines.xhtml

want their infant to be randomised and enrolled in the trial. Parents will be approached shortly after their infant is admitted to the neonatal unit (in most cases within the first 24 hours). There is no upper time limit as to when trial discussions can take place. Parents will be able to opt out of the WHEAT trial at any point. Neonatal health professionals will be prompted within the EPR to explain WHEAT to parents of eligible infants and to provide them with an information leaflet. If parents "opt out" this will be recorded in the EPR. If parents do not "opt out" and are happy for their infant to take part in WHEAT, randomisation will occur through the EPR. Enrolment of the infant and the allocation will be notified to the local team through the EPR. Because of the opt-out nature of WHEAT there will not be a signed consent form.

A qualitative exploration of the opt-out consent and recruitment process, and trial procedures will be conducted following the end of recruitment. Qualitative interviews will be undertaken with both parents that consented to the trial and health professionals from the recruiting sites.

Due to the common nature of packed red cell transfusion in the trial population (infants born at <30+0 gestational weeks+days), health professionals will explain the WHEAT trial and opt-out process shortly after birth (in most cases within the first 24 hours). A minority of infants will not receive a packed red cell transfusion during their neonatal unit stay. These will not be included in the main analysis population of clinical outcomes.

Results will be presented at national and international academic conferences and published in peer-reviewed scientific publications. Protocol author HR will work with the neonatal charity Bliss to produce parent-centred information for dissemination through social media, online and to be distributed on neonatal units.

For peer review only - http://bmjopen.bmj.com/site/about/guidelines.xhtml

Discussion

Preventing NEC is a recognised research priority in preterm birth [5], however there are no preventative interventions supported by high quality evidence. One key reason is because NEC is a rare condition, therefore any trial seeking to detect a realistic reduction in NEC will require recruitment and randomisation of more preterm infants than ever previously achieved. For example, a trial seeking to detect a 25% relative risk reduction in NEC from a background rate of 6% [18] would need to randomise over 9,000 infants to have 90% power to detect such a difference with a two-sided 5% significance level. The largest previous individually randomised trial that included preterm infants was the INIS trial [23] which enrolled 3,493 infants. Undertaking neonatal trials on this scale will be challenging; for such large trials to be funded and sustainable, they will need to be more efficient, less burdensome and international in scope. There are successful examples of large simple trials in other specialties that can inform neonatal practice: the TASTE trial [24] demonstrated high efficiency and low burden by integrating trial processes within an existing data capture system, and the TRANSFUSE trial [25] demonstrated very high recruitment rates (>75%) through the use of opt-out models of consent. The WHEAT pilot trial will apply these approaches and measure their feasibility and acceptability in neonatal care. If these methodologies can be successfully applied, they will facilitiate efficient, large, simple trials suitable to address the many clinical uncertainties the plague neonatal care [26].

The WHEAT pilot trial will determine the feasibility of addressing an important clinical question regarding the optimal approach to feeding preterm infants around the time of red cell transfusions, in preparation for a future definitive trial. Currently there is

For peer review only - http://bmjopen.bmj.com/site/about/guidelines.xhtml

insufficient evidence to recommend withholding or continuing milk feeds around red cell transfusion in preterm infants because available physiological and observational data are inconclusive.

Strength and limitations

The proposed trial has a number of strengths. The robustness of core NNRD data (birth weight, sex, length of stay and death) have been previously demonstrated for research purposes [1, 27, 28], this pilot trial will prospectively evaluate their accuracy and completeness for clinical trials. The trial will evaluate the feasibility of recruiting infants across two neonatal networks, including smaller neonatal units that do not traditionally recruit into neonatal randomised trials. Limitations include the unblinded nature of the trial and the use of a potentially subjective primary outcome, NEC. We endeavoured to mitigate against these through use of a previously validated, objective definition for NEC [1].

Conclusion

Neonatal trials to date have been unable to robustly evaluate strategies to prevent major preterm morbidities, such as optimal feeding around transfusion to prevent NEC, because of the large sample sizes required. This protocol describes a prospective, randomised controlled pilot trial to evaluate trial methodologies aiming to efficiently address such neonatal uncertainties.

Abbreviations

BSI blood stream infections

For peer review only - http://bmjopen.bmj.com/site/about/guidelines.xhtml

HQIP Healthcare Quality Improvement Partnership

NDAU Neonatal Data Analysis Unit

NEC necrotising enterocolitis

NNAP National Neonatal Audit Programme

NNRD National Neonatal Research Database

UK United Kingdom

UKNC United Kingdom Neonatal Collaborative

Consent for publication

Not applicable as the manuscript does not include any individual details.

Availability of data and material

The database to be used in this study is the NNRD which is managed at the Neonatal Data Analysis Unit directed by Professor Neena Modi; researchers, clinicians, managers, commissioners, and others are welcome and encouraged to utilise the NNRD. More details are available here: http://www.imperial.ac.uk/neonatal-data-analysis-unit/neonatal-data/utilising-the-nnrd/

Competing interests

The authors declare that they have no competing interests.

Funding

For peer review only

The trial is funded through a United Kingdom Medical Research Council (MRC) Clinician Scientist Fellowship awarded to CG.

Authors contributions

CG, NM, JD, AF, MT, HR, TvS and CG conceived the study; CG, NM, SJ, LC, JD, UB, AF, AK, JM, LL, MT, HR, KS, TvS and EJ contributed to the planning, conduct, and reporting of the study, and writing this manuscript. All authors read and approved the final manuscript. HR is a parent of a preterm twins and LC is a representative of Bliss the charity for babies born premature or sick.

Acknowledgements

We are grateful to all the families that agreed to the inclusion of their baby's data in the NNRD, the health professionals who recorded data and the NDAU team, the members of the study steering group, and the members of the NU Neonatal Collaborative representing neonatal units that contribute data to the NNRD.

References

1. Battersby C, Longford NT, Mandalia S, Costeloe K, Modi N: **Incidence and enteral feed antecedents of severe neonatal necrotising enterocolitis across neonatal networks in England, 2012–13: a whole-population surveillance study**. *The Lancet Gastroenterology & Hepatology* 2017, **2**(1):43-51.
2. Duro D, Kalish LA, Johnston P, Jaksic T, McCarthy M, Martin C, Dunn JC, Brandt M, Nobuhara KK, Sylvester KG *et al*: **Risk factors for intestinal failure in infants with necrotizing enterocolitis: a Glaser Pediatric Research Network study**. *J Pediatr* 2010, **157**(2):203-208 e201.
3. Hintz SR, Kendrick DE, Stoll BJ, Vohr BR, Fanaroff AA, Donovan EF, Poole WK, Blakely ML, Wright L, Higgins R *et al*: **Neurodevelopmental and growth outcomes of extremely low birth weight infants after necrotizing enterocolitis**. *Pediatrics* 2005, **115**(3):696-703.
4. Rees CM, Pierro A, Eaton S: **Neurodevelopmental outcomes of neonates with medically and surgically treated necrotizing enterocolitis**. *Arch Dis Child Fetal Neonatal Ed* 2007, **92**(3):F193-198.
5. Duley L, Uhm S, Oliver S, Preterm Birth Priority Setting Partnership Steering G: **Top 15 UK research priorities for preterm birth**. *Lancet* 2014, **383**(9934):2041-2042.

For peer review only

For peer review only - http://bmjopen.bmj.com/site/about/guidelines.xhtml

6. Seges RA, Kenny A, Bird GW, Wingham J, Baals H, Stauffer UG: **Pediatric surgical patients with severe anaerobic infection: report of 16 T-antigen positive cases and possible hazards of blood transfusion**. *Journal of pediatric surgery* 1981, **16**(6):905-910.

7. Stritzke AI, Smyth J, Synnes A, Lee SK, Shah PS: **Transfusion-associated necrotising enterocolitis in neonates**. *Arch Dis Child Fetal Neonatal Ed* 2013, **98**(1):F10-14.

8. Blau J, Calo JM, Dozor D, Sutton M, Alpan G, La Gamma EF: **Transfusion-related acute gut injury: necrotizing enterocolitis in very low birth weight neonates after packed red blood cell transfusion**. *J Pediatr* 2011, **158**(3):403-409.

9. Mally P, Golombek SG, Mishra R, Nigam S, Mohandas K, Depalhma H, LaGamma EF: **Association of necrotizing enterocolitis with elective packed red blood cell transfusions in stable, growing, premature neonates**. *Am J Perinatol* 2006, **23**(8):451-458.

10. Cunningham KE, Okolo FC, Baker R, Mollen KP, Good M: **Red blood cell transfusion in premature infants leads to worse necrotizing enterocolitis outcomes**. *J Surg Res* 2017, **213**:158-165.

11. El-Dib M, Narang S, Lee E, Massaro AN, Aly H: **Red blood cell transfusion, feeding and necrotizing enterocolitis in preterm infants**. *J Perinatol* 2011, **31**(3):183-187.

12. Sahin S, Gozde Kanmaz Kutman H, Bozkurt O, Yavanoglu Atay F, Emre Canpolat F, Uras N, Suna Oguz S, Underwood MA: **Effect of withholding feeds on transfusion-related acute gut injury in preterm infants: a pilot randomized controlled trial**. *J Matern Fetal Neonatal Med* 2019:1-6.

13. Jasani B, Rao S, Patole S: **Withholding Feeds and Transfusion-Associated Necrotizing Enterocolitis in Preterm Infants: A Systematic Review**. *Adv Nutr* 2017, **8**(5):764-769.

14. Parige R, Turner C, Sundaram S, Power S: **Enteral feeding during packed red blood cell transfusion in English neonatal units**. *Arch Dis Child Fetal Neonatal Ed* 2013.

15. Gale C, Hyde MJ, Modi N, group Wtd: **Research ethics committee decision-making in relation to an efficient neonatal trial**. *Arch Dis Child Fetal Neonatal Ed* 2016.

16. van Staa TP, Dyson L, McCann G, Padmanabhan S, Belatri R, Goldacre B, Cassell J, Pirmohamed M, Torgerson D, Ronaldson S *et al*: **The opportunities and challenges of pragmatic point-of-care randomised trials using routinely collected electronic records: evaluations of two exemplar trials**. *Health Technol Assess* 2014, **18**(43):1-146.

17. Battersby C, Longford N, Costeloe K, Modi N, Group UKNCNES: **Development of a Gestational Age-Specific Case Definition for Neonatal Necrotizing Enterocolitis**. *JAMA Pediatr* 2017.

18. RCPCH: **National Neonatal Audit Programme (NNAP) 2018 annual report on 2017 data**. In. London: Royal College of Paediatrics and Child Health (RCPCH); 2018.

19. Digital N: **National Neonatal Data Set**. In. Edited by Dictionary ND, 3 edn. http://www.datadictionary.nhs.uk/web_site_content/navigation/national_neonatal_data_sets_menu.asp; 2016.

20. Spencer A, Modi N: **National neonatal data to support specialist care and improve infant outcomes**. *Archives of Disease in Childhood - Fetal and Neonatal Edition* 2012.

21. Bernardo J, Nowacki A, Martin R, Fanaroff JM, Hibbs AM: **Multiples and parents of multiples prefer same arm randomization of siblings in neonatal trials**. *J Perinatol* 2015, **35**(3):208-213.

22. Webbe J, Brunton G, Ali S, Duffy JMN, Modi N, Gale C: **Developing, implementing and disseminating a core outcome set for neonatal medicine**. *BMJ Paediatrics Open* 2017, **1**(e000048).

23. Group IC, Brocklehurst P, Farrell B, King A, Juszczak E, Darlow B, Haque K, Salt A, Stenson B, Tarnow-Mordi W: **Treatment of neonatal sepsis with intravenous immune globulin**. *N Engl J Med* 2011, **365**(13):1201-1211.

24. Frobert O, Lagerqvist B, Olivecrona GK, Omerovic E, Gudnason T, Maeng M, Aasa M, Angeras O, Calais F, Danielewicz M *et al*: **Thrombus aspiration during ST-segment elevation myocardial infarction**. *N Engl J Med* 2013, **369**(17):1587-1597.

For peer review only

For peer review only - http://bmjopen.bmj.com/site/about/guidelines.xhtml

25. Cooper DJ, McQuilten ZK, Nichol A, Ady B, Aubron C, Bailey M, Bellomo R, Gantner D, Irving DO, Kaukonen KM *et al*: **Age of Red Cells for Transfusion and Outcomes in Critically Ill Adults**. *N Engl J Med* 2017, **377**(19):1858-1867.

26. Willhelm C, Girisch W, Gottschling S, Graber S, Wahl H, Meyer S: **Systematic Cochrane reviews in neonatology: a critical appraisal**. *Pediatr Neonatol* 2013, **54**(4):261-266.

27. Gale C, Santhakumaran S, Nagarajan S, Statnikov Y, Modi N: **Impact of managed clinical networks on NHS specialist neonatal services in England: population based study**. *BMJ* 2012, **344**:e2105.

28. Battersby C, Statnikov Y, Santhakumaran S, Gray D, Modi N, Costeloe K, Collaborative UKN, Medicines for Neonates Investigator G: **The United Kingdom National Neonatal Research Database: A validation study**. *PLoS One* 2018, **13**(8):e0201815.

For peer review only

The WHEAT pilot trial

WithHolding Enteral feeds Around packed red cell Transfusion to prevent necrotising enterocolitis in preterm neonates: a multi-centre, electronic patient record (EPR), randomised controlled point-of-care pilot trial

V2.0 26/07/2018

SPONSOR: Imperial College London
SPONSOR REFERENCE: 181C4529
FUNDERS: Medical Research Council (MR/N008405/1)
TRIAL COORDINATION CENTRE: National Perinatal Epidemiology Unit Clinical Trials Unit (NPEU CTU)
REC reference: 18/LO/0900
ISRCTN registration number: 62501859
IRAS ID: 154432

Chief Investigator: Dr Chris Gale
Trial Statistician: Sena Jawad, Imperial College London

Trial Coordination Centre

For general queries and supply of trial documentation please contact:

Trial Manager: Kayleigh Stanbury
Address: NPEU Clinical Trials Unit
National Perinatal Epidemiology Unit
Nuffield Department of Population Health
University of Oxford, Old Road Campus
Oxford, OX3 7LF
Tel: 01865 617923
Email: kayleigh.stanbury@npeu.ox.ac.uk
Fax: 01865 289740
Web address: http://www.npeu.ox.ac.uk/WHEAT

For peer review only

Clinical Queries

Clinical queries should be directed to Dr Chris Gale (CI) who will direct the query to the appropriate person:

	Dr Chris Gale
Address:	Neonatal Medicine
	Chelsea and Westminster Hospital Campus
	4th floor, lift bank D
	369 Fulham Road
	London, SW10 9NH
Tel:	0203 315 3519
Email:	christopher.gale@imperial.ac.uk

Sponsor

Imperial College London is the research Sponsor for this trial. For further information regarding the sponsorship conditions, please contact the Head of Regulatory Compliance at:

	Joint Research Compliance Office
	Imperial College London & Imperial College Healthcare NHS Trust
	2nd Floor Medical School Building
	St Mary's Hospital
	Praed Street
	London, W2 1NY
Tel:	0207 594 1872

Funder

Medical Research Council (UK), reference: MR/N008405/1

For peer review only - http://bmjopen.bmj.com/site/about/guidelines.xhtml

TABLE OF CONTENTS

Glossary of Abbreviations 5
Trial Summary 6
WHEAT Trial Flowchart 8
1. INTRODUCTION 9
- **1.1. BACKGROUND** 9
- **1.2. RATIONALE FOR CURRENT TRIAL** 11

2. TRIAL OBJECTIVES 12
3. TRIAL DESIGN 13
- **3.1. OVERALL DESIGN** 13
- **3.2. DURATION** 14
- **3.3. GEOGRAPHICAL AREA** 14

4. PARTICIPANTS 14
- **4.1. INCLUSION CRITERIA** 14
- **4.2. EXCLUSION CRITERIA** 14
- **4.3. WITHDRAWAL CRITERIA** 14
- **4.4. SETTING** 15
- **4.5. INTER-HOSPITAL TRANSFER** 15
- **4.6. END OF TRIAL** 15

5. PATHWAYS OF CARE TO BE COMPARED 15
- **5.1. PATHWAYS OF CARE** 15
- **5.2. CONCOMITANT CARE** 17

6. TRIAL OUTCOME MEASURES 17
- **6.1. FEASIBILITY OUTCOMES** 17
- **6.2. CLINICAL OUTCOMES** 18

7. RANDOMISATION AND ENROLMENT PROCEDURE 18
- **7.1. RANDOMISATION OR REGISTRATION PRACTICALITIES** 18
- **7.2. CONSENT** 19
- **7.3. RANDOMISATION** 19
- **7.4. BLINDING** 20

8. ADVERSE EVENTS 20
- **8.1. DEFINITIONS** 20
- **8.2. REPORTING PROCEDURES** 21

For peer review only

For peer review only - http://bmjopen.bmj.com/site/about/guidelines.xhtml

9. ASSESSMENT AND FOLLOW-UP 22
9.1. DATA COLLECTION BEFORE DISCHARGE 22
10. STATISTICS AND DATA ANALYSIS 22
10.1. DESCRIPTIVE STATISTICS 23
10.2. COMPARATIVE STATISTICS 23
11. MONITORING 23
11.1. RISK ASSESSMENT 23
11.2. MONITORING AT TRIAL COORDINATING CENTRE 23
11.3. MONITORING AT LOCAL SITE 23
12. REGULATORY ISSUES 24
12.1. ETHICS APPROVAL 24
12.2. CONFIDENTIALITY 24
12.3. INDEMNITY 24
12.4. SPONSOR 24
12.5. FUNDING 24
12.6. AUDITS 24
13. TRIAL MANAGEMENT 25
14. PUBLICATION POLICY 25
15. REFERENCES 26
Record of changes 30
APPENDIX 1 - Summary of investigations, treatment and assessments 31

For peer review only

For peer review only - http://bmjopen.bmj.com/site/about/guidelines.xhtml

GLOSSARY OF ABBREVIATIONS

AE	Adverse Event
ANCOVA	Analysis of covariance
CI	Chief Investigator
CTU	Clinical Trials Unit
DMC	Data Monitoring Committee
EPR	Electronic Patient Record
GCP	Good Clinical Practice
HRA	Health Research Authority
ISRCTN	International Standard Randomised Controlled Trial Number
NEC	Necrotising enterocolitis
NHS R&D	National Health Service Research & Development
NNAP	National Neonatal Audit Programme
NNRD	National Neonatal Research Database
NPEU	National Perinatal Epidemiology Unit
NRES	National Research Ethics Service
PMG	Project Management Group
REC	Research Ethics Committee
SAE	Serious Adverse Event
SDS	Standard Deviation Score
SHOT	Serious Hazards of Transfusion
SOP	Standard Operating Procedure
TAMBA	Twins And Multiple Births Association
TSC	Trial Steering Committee
UK	United Kingdom
USA	United States of America

For peer review only

For peer review only - http://bmjopen.bmj.com/site/about/guidelines.xhtml

TRIAL SUMMARY

**TITLE**	WithHolding Enteral feeds Around packed red cell Transfusion to prevent necrotising enterocolitis in preterm neonates: a multi-centre, electronic patient record (EPR), randomised controlled point-of-care pilot trial
**DESIGN**	A randomised, controlled, unblinded, pragmatic, superiority pilot trial, embedded within an electronic patient record system (point-of-care trial), comparing two parallel care pathways
**AIMS**	Pilot trial objective: To demonstrate the feasibility and efficiency of a point-of-care trial approach embedded within an electronic patient record (EPR) system<br><br>Clinical objective of planned main trial: To test whether the practice of withholding enteral feeds around packed red cell transfusion in preterm infants reduces the incidence of severe necrotising enterocolitis
**POPULATION**	Preterm infants (born less than 30^{+0} gestational weeks^{+days}) admitted to participating UK neonatal units
**ELIGIBILITY**	Inclusion criteria:<br>• Preterm birth at less than 30^{+0} gestational weeks^{+days} (up to and including 29^{+6} gestational weeks^{+days})<br><br>Exclusion criteria:<br>• Packed red cell transfusion with concurrent enteral feeds prior to enrolment (infants who have received a packed red cell transfusion while nil by mouth ARE still eligible)<br>• Infants where enteral feeding is contraindicated in the first 7 days after birth (e.g. congenital abnormalities)
**CARE PATHWAYS TO BE COMPARED**	1. WITHHOLD FEEDS AROUND TRANSFUSION: All enteral feeds will be discontinued (the infant will be placed nil by mouth) for a period of 4 hours prior to the transfusion, during the transfusion and until 4 hours post transfusion<br>2. CONTINUE FEEDS AROUND TRANSFUSION: Continuation of enteral feeding before, during and after transfusion<br><br>The same allocated care pathway will be followed for all transfusions a participating infant receives until and including 34^{+6} gestational weeks^{+days} or discharge (if sooner)
**OUTCOME MEASURES**	Pilot trial endpoints:<br>• Recruitment rate<br>• Opt-out rate<br>• Retention rate<br>• Compliance<br>• Data completeness of clinical endpoint data items<br>• Data accuracy of clinical endpoint data items

For peer review only - http://bmjopen.bmj.com/site/about/guidelines.xhtml

	Secondary (clinical) endpoints:<br>• Severe necrotising enterocolitis (surgically or histologically confirmed or recorded on death certificate)<br>• Spontaneous intestinal perforation surgically or histologically confirmed or recorded on death certificate)<br>• All-cause mortality<br>• Total duration of neonatal care (days)<br>• Duration of any parenteral nutrition (days)<br>• Length of time with a central venous line in situ (days)<br>• Number of central line associated blood stream infections (defined as per National Neonatal Audit Programme, NNAP, 2017 definition)<br>• Growth: change in weight and head circumference for gestational age standard deviation score between birth and final neonatal discharge
**DURATION**	Follow-up and evaluation of outcomes will be up to and including 40^{+0} gestational weeks^{+days} or neonatal unit discharge (if earlier).

For peer review only

For peer review only - http://bmjopen.bmj.com/site/about/guidelines.xhtml

WHEAT

WHEAT TRIAL FLOWCHART

Inclusion criteria

- Gestation at birth <30^{+0} weeks^{+days}
- Parents did not opt out of trial

Exclusion criteria

- Previous packed red cell transfusion with enteral feeds
- Enteral feeding contraindicated in first 7 days after birth

Randomisation (1:1 allocation ratio)

Secure online randomisation integrated into electronic patient record system (BadgerNet or BadgerEPR)

For each individual infant

WITHHOLD FEEDS AROUND TRANSFUSION FOR 4 HOURS BEFORE, DURING AND FOR 4 HOURS AFTER

CONTINUE FEEDS AROUND TRANSFUSION BEFORE, DURING AND AFTER TRANSFUSION

The allocated care pathway (transfusion feeding practice) should be applied around all packed red cell transfusions up to and including 34^{+6} gestational weeks^{+days} or discharge (if sooner)

All trial data will be extracted from the electronic clinical summary system up to and including 40^{+0} gestational weeks^{+days} or neonatal unit discharge (if sooner)

Feasibility outcomes

- Recruitment rate
- Opt-out rate
- Retention rate
- Compliance
- Data completeness
- Data accuracy

Clinical outcomes

- Severe necrotising enterocolitis (surgically or histologically confirmed or recorded on death certificate)
- Spontaneous intestinal perforation (surgically or histologically confirmed or recorded on death certificate), all-cause mortality, length of neonatal unit stay, duration of any parenteral nutrition, central venous line days, central line associated blood stream infections, change in weight and head circumference, standard deviation score between birth and final neonatal discharge

For peer review only - http://bmjopen.bmj.com/site/about/guidelines.xhtml

1. INTRODUCTION

1.1. BACKGROUND

Necrotising enterocolitis (NEC) is among the most devastating of neonatal diseases. UK population data from National Neonatal Research Database (NNRD) indicates that severe NEC (requiring surgery or resulting in death) affects about 5% of infants born at less than 30 gestational weeks, and has a mortality of about 33% (1); NEC is also a major cause of long-term gastrointestinal morbidity and the leading cause of paediatric short bowel syndrome (2). The inflammatory process extends the effects of the disease systemically, and affected infants are at substantially increased risk of neurodevelopmental impairment (3, 4). In England over 2012–13, 531 infants developed severe NEC and one third of these died of the disease (1).

A temporal association between red cell transfusion and the subsequent development of NEC was originally described in the 1980s (5), and continues to be described in observational studies (6). In comparison with classical NEC, transfusion associated NEC cases are anecdotally described as more severe (7) with higher rates of surgical intervention (8) and higher mortality (9, 10).

1.1.1. Proposed mechanisms linking transfusion and necrotising enterocolitis

The pathogenesis of NEC is not completely understood. It is believed that NEC arises from "an uncontrolled exuberant inflammatory response to bacterial colonization that characterises the intestine of the preterm infant" (11). The innate immune system, and specifically up-regulation of toll like receptor 4, mediate this inflammatory response (12), while altered commensal intestinal microbiota and impaired intestinal epithelial integrity are contributory. Factors that are believed to increase an infant's risk of NEC are those that alter the commensal intestinal flora (such as prolonged treatment with antibiotics, absence of human milk feeds), or impair mucosal integrity (such as prolonged absence of milk or formula feeding, or profound hypotension) (13).

Milk feeds during packed red cell transfusion may precipitate NEC by influencing mesenteric blood flow and thus intestinal barrier function. Mesenteric blood flow is increased in response to milk feeds (14, 15), and absence of this normal postprandial increase is seen in infants who subsequently develop NEC (16), leading to speculation that this relative gut hypoperfusion may predispose infants to NEC (16, 17).

Packed red cell transfusion results in a failure of the normal postprandial increase in mesenteric blood flow in preterm lamb (18) and piglet models (19), and in human preterm infants (7, 17, 20, 21). The cessation of milk feeds around the time of packed red cell transfusion may therefore be beneficial in limiting the influence on intestinal blood flow (22). This practice has not, however, been tested in a published randomised trial.

1.1.2. Potential adverse effects of interrupting milk feeds

Conversely, stopping milk feeds around blood transfusions among preterm infants at high risk of necrotising enterocolitis, may lead to harm. Interrupting milk feeding may prolong the time taken to reach full enteral feeds, which is associated with increased risk of invasive

For peer review only - http://bmjopen.bmj.com/site/about/guidelines.xhtml

infection (23). Furthermore, in preterm infants, a higher number of days where feeds are withheld is associated with an increased risk of necrotising enterocolitis (24), raising the possibility that the intervention proposed to reduce transfusion associated necrotising enterocolitis (withholding milk feeds) may, in fact, lead to the very disease it is aiming to prevent.

1.1.3. Red cell transfusion in preterm infants

Preterm infants are among the most transfused patient groups; 90–95% of infants born at <30 weeks of gestation receive at least one blood transfusion (25); those transfused received a mean of 4 (range 1–27) transfusions during their neonatal unit stay (population level data from NNRD). Of note is that randomised trials aiming to reduce the number of packed red cell transfusions received by preterm infants, though succeeding in this aim, did not show any associated reduction in NEC (26, 27).

1.1.4. Evidence from non-randomised studies

There have been no adequately powered randomised studies that have examined the question "Does withholding feeds during transfusion reduce the occurrence of transfusion associated NEC?"

Non-randomised studies were recently reviewed by Jasani et al in September 2017 (28). This systematic review identified seven non-randomised studies, including 7,492 infants. Included studies were historical control studies and were therefore at high risk of bias, including regression to the mean and ascertainment bias. Pooled results from the identified non-randomised studies suggest that withholding feeds during the peri-transfusion period may reduce the risk of transfusion associated necrotising enterocolitis in preterm infants. The authors conclude that adequately powered randomised controlled trials are needed to confirm these findings.

1.1.5. Current randomised studies

Review of clinical trial registries (WHO ICTRP, searched 1/8/2017) identified two single centre randomised controlled trials examining enteral feeding around blood transfusion in preterm infants. Neither trial is powered to examine clinically relevant outcomes such as NEC.

1. FEEding DURing Red Cell Transfusion (FEEDUR): The effects of feeding on blood flow to the gut in preterm infants receiving red blood cell transfusion; ANZCTR identifier ACTRN12616000160437, Newborn Care Centre Royal Hospital for Women, Sydney, Australia. The primary outcome is a non-clinical outcome, cerebro-splanchnic oxygenation ratio measured using near infrared spectroscopy. The planned sample size is 60 infants; the trial is still recruiting.
2. Tx-TRAGI trial, ClinicalTrials.gov identifier NCT02132819, Zekai Tahir Burak Maternity and Teaching Hospital, Turkey. The primary outcome is increase in abdominal circumference and NEC defined using Bell's staging criteria (29). The planned sample size is 150 infants; the trial is still recruiting.

For peer review only - http://bmjopen.bmj.com/site/about/guidelines.xhtml

1.1.6. Current practice

Considerable variation in current UK practice exists in relation to withholding enteral feeds during packed red cell transfusion in preterm infants, reflecting the limited evidence base for this approach. A 2011 electronic survey of UK neonatal units (68% response rate) demonstrated that 35% of UK units routinely withheld enteral feeds during packed red cell transfusion (30). We updated this survey in 2014 and found similar results; 106/163 neonatal units responded, 28% routinely withhold enteral feeds during transfusion, in 22% the decision to withhold feeds was left up to the individual clinician and 50% did not routinely withhold enteral feeds during transfusion. A survey carried out in the USA in 2009 recorded that 17% of American units practised withholding enteral feeds around blood transfusion (31).

1.1.7. Importance

If withholding enteral feeds around the time of packed red cell transfusion reduces the risk of NEC, then this simple practice will provide a way to reduce the mortality and long-term health and neurodevelopmental burden associated with this disease.

Conversely, given that human milk contains a number of growth factors and immunological agents, it is biologically plausible that episodes of withholding feeds in preterm might adversely affect intestinal integrity and development, and paradoxically increase the risk of NEC or poor growth. Given how widespread the practice of withholding enteral feeds is in the UK and internationally, demonstrating harm will mean that this practice can be safely discontinued.

1.1.8. Relevance

Prevention of NEC has been identified by service users and clinicians as the third most important treatment uncertainty in the field of preterm birth (32). The National Blood and Transplant Serious Hazards Of Transfusion (SHOT) report in 2012 and 2013 reported cases of NEC possibly associated with packed red cell transfusion and called for prospective studies to investigate a causal relationship (33, 34), and in 2016 transfusion associated NEC was identified as a research gap in transfusion medicine (35). There have been multiple published calls for a large scale randomised controlled trial from academics, clinicians (13, 20, 22, 36-39) and nursing professionals (40, 41).

1.2. RATIONALE FOR CURRENT TRIAL

1.2.1. Research question

Among preterm infants (Patient), does the practice of withholding enteral feeds around the time of blood transfusion (Intervention), compared with continued enteral feeding around the time of blood transfusion (Comparator), lead to a reduction in severe necrotising enterocolitis (Outcome)?

This is a pilot trial (not an internal pilot) to determine whether a large multi-centre trial addressing this research question is feasible, and whether clinical trial processes (identifying participants, randomisation and data collection) can be successfully integrated into existing

For peer review only - http://bmjopen.bmj.com/site/about/guidelines.xhtml

neonatal Electronic Patient Record (EPR) systems: the BadgerNet clinical summary system, and the BadgerEPR full electronic patient record system.

The clinical question that underpins the WHEAT trial is an important source of clinical uncertainty in the United Kingdom and internationally, and has been identified as an important research priority by multiple groups. Because the substantive clinical outcome (necrotising enterocolitis) is rare, a trial powered to detect a clinically important reduction would need to be very large: larger than any previous randomised controlled trial among preterm infants carried out anywhere in the world. It is for this reason that the WHEAT pilot trial is evaluating the feasibility of using an existing neonatal EPR system to apply a point-of-care trial methodology.

A point-of-care trial embeds trial processes, including the identification, recruitment, randomisation and trial data collection into an existing data collection structure, in this case an existing neonatal EPR (the BadgerNet clinical summary system and the BadgerEPR full electronic patient record system). Applying a point-of-care trial methodology offers the potential for large improvements in efficiency, and hence the possibility of studying much larger participant numbers. However, feasibility and data quality have not yet been demonstrated for the UK neonatal EPR systems (BadgerNet and BadgerEPR) for the purposes of a point-of-care trial.

It is these feasibility questions that the WHEAT pilot trial will address, in preparation for a future trial that will be powered to address the clinically important outcome necrotising enterocolitis.

2. TRIAL OBJECTIVES

	Objective	Outcomes
**Feasibility**	To determine whether a point-of-care trial methodology (embedding trial processes and data collecting within an existing EPR system) is feasible for an individually randomised trial that includes preterm infants delivered at less than 30^{+0} gestational $\text{weeks}^{+\text{days}}$, carried out in NHS neonatal units.<br><br>Outcomes will be measured up to and including 40^{+0} gestational $\text{weeks}^{+\text{days}}$ or neonatal unit discharge (if sooner).	Recruitment rate: Percentage of eligible cases where parents agree to trial involvement and the infant is randomised.<br><br>Opt-out rate: Percentage of eligible cases where parents opted out of their infant being involved in the trial.<br><br>Retention rate: Percentage of recruited infants who complete follow-up.<br><br>Compliance: Percentage of cases where the allocated care pathway was adhered to.<br><br>Data completeness: Percentage of recruited infants where trial data items are complete.

For peer review only - http://bmjopen.bmj.com/site/about/guidelines.xhtml

		Data accuracy: Percentage of recruited infants where data items are correctly recorded when compared to source data.
**Clinical**	To determine if withholding enteral feeds around blood transfusion is superior to continued enteral feeding, in reducing incidence of NEC and other clinical outcomes before discharge from neonatal care<br><br>Outcomes will be measured up to and including 40^{+0} gestational $weeks^{+days}$ or neonatal unit discharge (if sooner).	Necrotising enterocolitis: Histologically or surgically confirmed, or recorded in part 1 of the death certificate<br><br>Spontaneous intestinal perforation: Histologically or surgically confirmed, or recorded in part 1 of the death certificate<br><br>All-cause mortality<br><br>Length of neonatal unit stay: in days and including all levels of care<br><br>Duration of any parenteral nutrition: in days<br><br>Number of days with a central venous line in situ<br><br>Number of central line associated blood stream infections: defined according to NNAP criteria<br><br>Growth: change in weight and head circumference for gestational age standard deviation score between birth and final neonatal discharge

3. TRIAL DESIGN

3.1. OVERALL DESIGN

The WHEAT trial is a randomised, controlled, unblinded, multi-centre, superiority pilot trial of two care pathways. The primary metrics of feasibility are recruitment, data completeness and data accuracy. The clinical outcomes include mortality and NEC. Groups will be randomised with a 1:1 allocation ratio with varied block sizes and stratified within neonatal unit by gestational age at birth and infant sex. Trial processes will be embedded within neonatal EPR systems and all outcome data will be extracted from data that is routinely recorded within the existing neonatal EPR systems (BadgerNet and BadgerEPR), and held in the National Neonatal Research Database.

The WHEAT trial is a stand-alone pilot trial to demonstrate that the point-of-care methodology applied in WHEAT is efficient and results in complete and accurate trial data.

For peer review only - http://bmjopen.bmj.com/site/about/guidelines.xhtml

3.2. DURATION

Recruitment: 9 months

Randomised care pathway phase: Until all trial infants have finished the randomised care pathway (all enrolled infants are older than a gestational age of 34^{+6} gestational weeks^{+days})

Follow-up phase: Until all trial infants have finished the follow-up period (40^{+0} gestational weeks^{+days} or neonatal unit discharge, if earlier)

Number of infants to be recruited:

- Estimated number of eligible infants in 2 neonatal networks recruiting to the WHEAT trial: in the region of 375 (annual number of <30 gestational week infants cared for in the proposed trial networks in 2016 was 500, from NNRD data)
- Estimated recruitment rate of 65–70%
- Estimated recruitment over 9 months of 250

3.3. GEOGRAPHICAL AREA

The WHEAT trial will recruit patients from neonatal networks in England.

4. PARTICIPANTS

4.1. INCLUSION CRITERIA

1. Preterm birth at less than 30^{+0} gestational weeks^{+days} (up to and including 29^{+6} gestational weeks^{+days})
2. Parents did not opt out of trial participation

4.2. EXCLUSION CRITERIA

1. Packed red cell transfusion with concurrent enteral feeds prior to enrolment (infants who have received a packed red cell transfusion while nil by mouth ARE still eligible)
2. Infants where enteral feeding is contraindicated in the first 7 days after birth (e.g. congenital abnormalities)

Infants enrolled in other interventional studies are eligible for participation in the WHEAT trial unless contraindicated (Chief Investigators to discuss on a case-by-case basis).

Use of any concomitant medication used for neonatal clinical care or as part of an interventional research trial is permitted during the WHEAT trial.

4.3. WITHDRAWAL CRITERIA

If parents choose to withdraw their infant from receiving the allocated pathway of care, they will be asked for permission for continuing data collection and/or follow-up.

The attending clinician may withdraw the infant from the allocated pathway of care if they consider this to be in the best interest of the infant's health and well-being.

For peer review only - http://bmjopen.bmj.com/site/about/guidelines.xhtml

4.4. SETTING

Neonatal units in England caring for very preterm infants.

4.5. INTER-HOSPITAL TRANSFER

Participating neonatal units will be either:

1. A recruiting site where infants may be recruited, randomised, and commence participation in the trial
2. A continuing care site where the allocated care pathway (withhold feeds or feed as usual during transfusion) will continue to be followed and routine data collected if a participating infant is transferred in from a recruiting site

From recent experience, about 50% of participating infants are likely to be transferred from their recruiting neonatal care unit to a continuing care site.

4.6. END OF TRIAL

The trial will end when the last trial infant finishes follow-up (reaches 40^{+0} gestational $weeks^{+days}$ or neonatal unit discharge)

5. PATHWAYS OF CARE TO BE COMPARED

5.1. PATHWAYS OF CARE

Both comparator pathways of care are standard in the UK; there is no "experimental care pathway". The WHEAT trial is a comparative effectiveness trial. The two care pathways that will be compared are

1. Withholding feeds around transfusion
2. Continuing feeds around transfusion

Infants will remain allocated to the same care pathway until 34^{+6} $weeks^{+days}$ gestational age.

5.1.1. Withholding feeds around transfusion

Within the *withholding feeds around transfusion* pathway of care, all enteral feeds will be discontinued (the infant will be placed nil by mouth) for a period of 4 hours prior to packed red cell transfusion, during the packed red cell transfusion and until 4 hours post packed red cell transfusion.

During the period of this pathway of care (approximately 12 hours), hydration and blood glucose will be maintained according to local practice, commonly by provision of parenteral nutrition or intravenous dextrose.

Four hours after the red cell transfusion has finished feeds will be restarted in the manner in which they were being received prior to the decision to transfuse.

For peer review only - http://bmjopen.bmj.com/site/about/guidelines.xhtml

5.1.2. Continuing feeds around transfusion

Within the *continuing feeds around transfusion* pathway of care, enteral feeds will continue to be given prior, during and after the packed red cell transfusion, in the manner in which they were being given prior to the decision to transfuse.

5.1.3. Justification for the duration of the withholding feeds arm

Where withholding milk feeds around packed red cell transfusion is practised, there is no consensus regarding the duration of withholding feeds. The most recent UK data show that among neonatal units where milk feeds were routinely stopped for transfusion, 66% stopped feeds only for the duration of the transfusion and the remainder adjusted feeds for a variable period of time ranging from 4 hours before to 4 hours after transfusion (30). In the USA, among neonatal units where milk feeds are stopped, 82% withheld feeds before transfusion and 71% after (the duration was not recorded) (31).

Similar variation exists in studies where the association between withholding feeds and NEC has been examined using historical cohorts. DeRienzo et al (39) stopped enteral feeds 4 hours prior to and for the duration of a transfusion and restarted enteral feeds at 50% volume for 12 hours before advancing to the original volume of feeds. Feeds were omitted for 2–4 hours before and after as well as during packed red cell transfusions by Del Vecchio et al (42), and for 4 hours before and 4 hours after by Perciaccante et al (43), while in the study by El-Dib et al (22) feeds were omitted only for the duration of the transfusion. In the only registered randomised controlled trial (44) feeds are omitted from 4 hours prior until 24 hours post transfusion.

One rationale for withholding enteral feeds before packed red cell transfusion is that unless feeds are discontinued in advance, milk within the stomach will transit into the small intestine (the site most commonly affected by NEC) during packed red cell transfusion, potentially influencing gut haemodynamics. The median oro-caecal transit time (a measure of both gastric emptying and small intestinal transit time) in preterm infants (10 infants, median gestational age at birth 28.9 weeks, median age at examination 19 days) is 3.1 hours; therefore withholding feeds for 4 hours prior should result in passage of milk through the small intestine before transfusion (45).

The rationale for withholding feeds after packed red cell transfusion is less clear. Intestinal haemodynamic effects may persist following blood transfusion, as mesenteric blood flow remains reduced for at least four hours after transfusion in preterm infants with a haemodynamically significant patent ductus arteriosus (46), but the total duration is unclear: no significant differences are detectable by 48–96 hours post transfusion (17).

In the light of considerable variation in practice and incomplete scientific knowledge, a survey was undertaken of neonatal units within the UK Neonatal Collaborative (all 163 neonatal units in England). In total 122 neonatal units (75%) responded, of which 112 expressed an interest in taking part in the WHEAT trial. The most acceptable duration to withhold feeds was for 4 hours prior to transfusion (56% of responders) and for 4 hours following transfusion (60% of responders).

Combining data from available scientific studies, existing practice and clinician preference has led to the decision to define the *withholding feeds around transfusion* pathway of care

For peer review only - http://bmjopen.bmj.com/site/about/guidelines.xhtml

as "withholding milk feeds for 4 hours prior to, during, and for 4 hours following transfusion".

5.1.4. Justification for the continue feeds arm

In both the UK (30) and the USA (31) the most common practice is to continue milk feeds at the previous rate prior to, during and following transfusion, justifying this practice for the control group.

5.2. CONCOMITANT CARE

In order to ensure that this pragmatic trial is as generalisable as possible to current practice, blood transfusions will be administered when clinically indicated according to local packed red cell transfusion guidelines. Data will be collected about pre-transfusion haemoglobin level for trial participants.

Other concomitant care, including speed of increase of enteral feeds and choice of milk, for both the *withholding feeds around transfusion* pathway and the *continuing feeds around transfusion* pathway of care will be according to locally defined practice.

5.2.1. Feed intolerance

In situations where enteral feeds intolerance is manifest, in either the *withholding feeds around transfusion* pathway or the *continuing feeds around transfusion* pathway, during the period of packed red cell transfusion (for example, vomiting) management will be in accordance with clinical practice considered appropriate by the local clinical team.

6. TRIAL OUTCOME MEASURES

6.1. FEASIBILITY OUTCOMES

1. Recruitment rate: Rate and percentage of eligible cases where parents agree to trial involvement and the infant is randomised in the WHEAT trial
2. Opt-out rate: Percentage of eligible cases where parents opt out of their infant being involved in the trial
3. Retention rate: Rate and percentage of recruited cases where outcome data are available up to the end of the follow-up period (40^{+0} gestational $weeks^{+days}$ or neonatal unit discharge, if earlier)
4. Compliance: Rate and percentage of recruited cases who correctly received their allocated care pathway around all packed red cell transfusions between randomisation and a gestational age of 34^{+6} gestational $weeks^{+days}$
5. Data completeness: Percentage of eligible cases where trial data items are complete
6. Data accuracy: Percentage of recruited cases where the following data items are correctly recorded when compared to source data (clinical notes where available or electronic patient record data)
    a. Severe necrotising enterocolitis – histologically or surgically confirmed, or recorded in part 1 of the death certificate, all infants with this outcome will have source data verified. All infants recorded as being transferred to stand-

For peer review only - http://bmjopen.bmj.com/site/about/guidelines.xhtml

alone surgical units (e.g. Birmingham Children's Hospital or Great Ormond Street Hospital) will have their source data verified (false-positive rate).

b. All-cause mortality (all infants with this outcome will have source data verified).
c. All infants with necrotising enterocolitis recorded as a diagnosis (any diagnosis of necrotising enterocolitis, of any severity) will have their source data verified to ensure that they do not meet the criteria for severe necrotising enterocolitis or died prior to neonatal unit discharge (assessing false-negative rate).
d.

6.2. CLINICAL OUTCOMES

1. Severe necrotising enterocolitis: histologically or surgically confirmed, or recorded in part 1 the death certificate. These infants will be identified as described in (47), a process which will include infants recorded as being transferred for surgery
2. Spontaneous intestinal perforation: histologically or surgically confirmed, or recorded in part 1 the death certificate.
3. All-cause mortality
4. Total duration of neonatal care in days: including all levels of care (intensive care, high dependency care, special care and ordinary care)
5. Duration of any parenteral nutrition in days
6. Number of days with a central venous line in situ
7. Number of central line associated blood stream infections (defined according to National Neonatal Audit Programme (NNAP) 2017 definition)
8. Growth: change in weight and head circumference for gestational age standard deviation score between birth and final neonatal discharge

7. RANDOMISATION AND ENROLMENT PROCEDURE

7.1. RANDOMISATION OR REGISTRATION PRACTICALITIES

Potential participants will be identified through the existing neonatal EPR systems that are widely used across England; BadgerNet (a clinical summary system) or BadgerEPR (a complete electronic patient record system).

Baseline data for all infants admitted to neonatal units in the UK are routinely entered into the EPR *admission summary* as part of normal clinical care. These data are updated in real-time and held securely on BadgerNet and BadgerEPR servers.

In participating units, data entered electronically into the *admission summary* will be interrogated by the EPR platform in real time to identify and flag infants meeting the WHEAT trial inclusion criteria. When an infant in a participating unit meets the inclusion criteria, this will result in an electronic reminder appearing on the EPR platform at the participating unit. This "flag" will inform the health professional that the infant is eligible for the WHEAT trial and link to the parent information leaflet.

The EPR system will use data (neonatal unit, gestational age and sex) entered as part of the *admission summary* to stratify infants.

For peer review only - http://bmjopen.bmj.com/site/about/guidelines.xhtml

7.2. CONSENT

Because both the care pathways that are being compared are part of standard UK practice, WHEAT is using a simplified model of consent. This means that parents will have the WHEAT trial explained to them and will be asked to “opt out” if they do not want their infant to be randomised and enrolled in the trial.

Parents will be approached shortly after their infant is admitted to the neonatal unit (in most cases within the first 24 hours). There is no upper time limit as to when trial discussions can take place. Parents will be able to opt out of the WHEAT trial at any point.

Neonatal health professionals will be prompted within the EPR to explain WHEAT to parents of eligible infants and to provide them with an information leaflet. The EPR will subsequently ask the health care professional whether the WHEAT trial and the “opt-out” process have been fully explained to the parents. If parents “opt out” this will be recorded in the EPR. If parents do not “opt out”, i.e. are happy for their infant to take part in WHEAT, randomisation will occur through the EPR.

Enrolment of the infant and the allocation will be notified to the local team through the EPR. Enrolment can take place at any time during an infant’s neonatal stay providing they meet the inclusion criteria. At the point of randomisation, the trial CI and the Clinical Trials Unit will be automatically notified electronically. Participating infant data will be downloaded regularly to the Neonatal Data Analysis Unit (NDAU).

Because of the opt-out nature of WHEAT there will not be a signed consent form. Prior to randomisation the EPR will ask the health professional to confirm that the parents have been provided with the trial information, have had the trial explained to them, have had an opportunity to ask questions and have not expressed the wish to “opt out”. This will be recorded electronically within the EPR.

Due to the common nature of packed red cell transfusion in the trial population (infants born at <30^{+0} gestational weeks^{+days}), health professionals will explain the WHEAT trial and opt-out process shortly after birth (in most cases within the first 24 hours). A minority of infants will not receive a packed red cell transfusion during their neonatal unit stay (estimated to be <5% of eligible infants). These will not be included in the main analysis population of clinical outcomes.

7.3. RANDOMISATION

Infants will be randomly assigned to either pathway of care in a 1:1 allocation ratio as per a computer generated randomisation sequence (stratified by neonatal unit) using permuted blocks of various sizes with stratification by gestational age and infant sex within neonatal unit. The block sizes will not be disclosed to ensure allocation concealment.

Stratification will be by neonatal unit of enrolment and using the following categories:

1. Gestational age at birth
    - <28^{+0} weeks^{+days}
    - 28^{+0} to 29^{+6} weeks^{+days}
2. Infant sex

For peer review only - http://bmjopen.bmj.com/site/about/guidelines.xhtml

7.3.1. Randomisation of multiple births

Infants that are part of a multiple birth set (twins, triplets or higher order multiples) will be randomised as a multiple – i.e. they will all be allocated to the same pathway of care (withholding feeds around transfusion or feeding as usual around transfusion). This decision is based upon feedback from parent representatives, parent organisations including Bliss and TAMBA (Twins and Multiple Births Association) and international research involving parents and adult ex-preterm twins (48).

7.3.2. Allocation concealment

Infants will be randomised using an online secure central randomisation service which will be embedded into the existing neonatal EPR systems (BadgerNet and BadgerEPR). Randomisation will occur within the EPR to ensure allocation concealment. A unique identifier will be generated within the EPR for each infant to enable trial data to be extracted from routinely entered clinical data.

7.4. BLINDING

Because it is not possible to mask the different care pathways, the WHEAT trial will be unblinded.

8. ADVERSE EVENTS

Due to the nature of the patient population, neonates in intensive care, a high incidence of adverse events is foreseeable during their routine care and treatment. Consequently, only those adverse events identified as serious will be recorded for the trial.

8.1. DEFINITIONS

8.1.1. Serious Adverse Event (SAE)

Adverse events are defined as serious if they:

- Result in death
- Are life-threatening
- Require inpatient hospitalisation or prolongation of existing hospitalisation
- Result in persistent or significant disability/incapacity, or
- Are a congenital anomaly/birth defect

The term “life-threatening” refers to an event in which the participant was at risk of death at the time of the event; it does not refer to an event which hypothetically might have caused death if it were more severe. SAEs are to be reported from randomisation until the end of trial follow-up (40^{+0} gestational weeks^{+days} or neonatal unit discharge, if earlier).

For peer review only - http://bmjopen.bmj.com/site/about/guidelines.xhtml

8.2. REPORTING PROCEDURES

8.2.1. Recording SAEs

Non-serious adverse events will not be routinely recorded as the trial is comparing two accepted pathways of care that are both widely practised in the United Kingdom.

8.2.2. Reporting foreseeable SAEs

The following are serious adverse events that could be reasonably anticipated to occur in this population of infants during the course of the trial or form part of the outcome data. They do not require reporting by the trial sites as SAEs but do require relevant data to be captured in the summary EPR systems (BadgerNet or BadgerEPR) as part of routine clinical care:

- Death (unless cause not anticipated in this population)
- Necrotising enterocolitis or gastrointestinal perforation
- Bronchopulmonary dysplasia (or chronic lung disease)
- Intracranial abnormality (haemorrhage or focal white matter damage) on cranial ultrasound scan or other imaging
- Pulmonary haemorrhage
- Pneumothorax
- Anaemia requiring blood transfusion
- Hyperbilirubinaemia
- Hyperglycaemia
- Hypoglycaemia
- Coagulopathy requiring treatment
- Hypotension
- Hypertension
- Impaired renal function
- Patent ductus arteriosus (PDA)
- Retinopathy of prematurity
- Sepsis
- Fractures
- Clinically significant liver failure
- Clinically significant extravasation injury
- Clinically significant left ventricular hypertrophy on echocardiography
- Hydrocephalus

Only if these events are thought to be causally related to the allocated pathway of care would they require urgent reporting to the trial centre as outlined below.

Unforeseen SAEs and the SAEs associated with the allocated pathway of care must be reported to the NPEU CTU by a member of site staff within 24hours of becoming aware of the event. Site staff may email or fax a completed paper SAE form to NPEU CTU. Paper forms, with instructions, will be made available with the trial documentation to enable anyone to report an SAE. If this is not possible, site staff may report the SAE to NPEU CTU by telephone and will follow up this notification with an SAE report form by fax or email as

For peer review only - http://bmjopen.bmj.com/site/about/guidelines.xhtml

soon as possible. If following the reporting of an SAE additional information becomes available, a new SAE form should be completed.

The NPEU CTU will forward a copy of the SAE form to the Chief Investigator (CI) as soon as possible on receipt. The CI will assess whether the SAE was as a result of trial related activities (related). All related and unexpected SAEs will be submitted to the Research Ethics Committee (REC) that gave a favourable opinion of the trial within 15 working days of the CI becoming aware of the event, using the HRA report of serious adverse event form (see HRA website). In addition, all unforeseen SAEs and the SAEs associated with the allocated pathway of care will be reported to the sponsor and related and unforeseeable SAEs will be reported to the DMC and relevant R&D offices.

Contact details for reporting SAEs

Fax: 01865 289740, Email: WHEAT@npeu.ox.ac.uk

Please scan and email or fax SAE forms to the WHEAT Trial Coordinating Centre

Tel: 01865 617923 (Mon to Fri 09.00–17.00)

9. ASSESSMENT AND FOLLOW-UP

Follow-up will be until neonatal unit discharge or 40^{+0} gestational $weeks^{+days}$, whichever is first. There will be no data collection after neonatal unit discharge.

9.1. DATA COLLECTION BEFORE DISCHARGE

All outcome data for this trial are routinely recorded clinical items held in the patient notes and existing neonatal EPR systems (BadgerNet and BadgerEPR). No additional blood or tissue samples are required for this trial.

Clinical information will be extracted from routinely recorded clinical data entered at the point of care by health professionals into the existing EPR (BadgerNet or the BadgerEPR).

10. STATISTICS AND DATA ANALYSIS

There is no predefined sample size for this pilot trial. Recruitment (absolute numbers and the rate) will be a primary outcome for the pilot trial. The estimated target sample size for the pilot trial is up to 250 based on infant throughput and assuming 65–70% recruitment of eligible infants in the neonatal networks.

As this is a pilot trialfocusing on feasibility outcomes rather than clinical outcomes, no formal sample size calculation was conducted and the target recruitment was estimated based on practical and realistic assumptions. The pilot trial aims to provide inference for the sample size calculation of the main trial.

As this is a pilot trial, the sample size is not powered to detect any treatment differences; therefore, no formal hypothesis testing will be conducted.

For peer review only - http://bmjopen.bmj.com/site/about/guidelines.xhtml

10.1. DESCRIPTIVE STATISTICS

All baseline and feasibility outcomes will be analysed descriptively. All continuous and normally distributed data will be presented as means and standard deviations and non-normally distributed data will be presented by medians along with 25th and 75th centiles and minimum and maximum values. All categorical and dichotomous variables will have frequencies and percentages presented.

10.2. COMPARATIVE STATISTICS

Feasibility results, including recruitment, data quality and data completeness will be reported as rates or proportions with 95% confidence intervals.

Although no formal hypothesis testing will be conducted, estimated differences in clinical outcomes of efficacy will be calculated.

Data and all essential documentation will be stored for a minimum of 25 years after the completion of the trial, including the follow-up period.

11. MONITORING

11.1. RISK ASSESSMENT

Prior to trial commencement, the NPEU CTU performed a risk assessment of the trial that will be reviewed at regular intervals according to its own Standard Operating Procedure. This trial is a comparison of standard treatments, which does not include a drug treatment, so does not fall under the auspices of the MHRA. Based on the assessment, this trial poses minimal risk, no greater than normal care within a neonatal intensive care unit, to either the participants or the health care professionals delivering the trial.

11.2. MONITORING AT TRIAL COORDINATING CENTRE

Central monitoring will be used at NPEU CTU to monitor patterns of recruitment at sites and within the data; data completeness and quality; safety reports and outliers in the clinical data will be investigated and may trigger 'for cause' site monitoring.

11.3. MONITORING AT LOCAL SITE

Direct access will be granted to authorised representatives from trial organisers, the research Sponsor and NHS Trusts to permit trial-related monitoring, audits and inspections.

Trial data accuracy and completeness are outcomes for this pilot trial. Source data verification will be undertaken by the WHEAT trial team for clinical outcome data items, stratification data items, and allocation data items as outlined in section 3.1, trial outcome measures.

For peer review only - http://bmjopen.bmj.com/site/about/guidelines.xhtml

12. REGULATORY ISSUES

12.1. ETHICS APPROVAL

The trial will only start after gaining approval from the Health Research Authority (HRA), and a National Research Ethics Service (NRES) registered ethics committee. Additionally, NHS Trust Research and Development (R&D) Offices will review the trial for Capacity and Capability for individual trial sites. The CI or their delegate will submit and, where necessary, obtain approval from the REC for any protocol amendments and changes to the parent information leaflet.

The trial will be conducted in accordance with the recommendations for physicians involved in research on human subjects adopted by the 18th World Medical Assembly, Helsinki 1964 and later revisions. This trial will adhere to the principles outlined in the NHS UK Policy Framework for Health and Social Care Research. It will be conducted in compliance with the protocol, relevant Data Protection regulations, the principles of GCP and other regulatory requirements as appropriate.

12.2. CONFIDENTIALITY

The Chief Investigator will preserve the confidentiality of participants taking part in the trial and is registered under relevant Data Protection regulations.

12.3. INDEMNITY

Imperial College London holds negligent harm and non-negligent harm insurance policies which apply to this trial.

12.4. SPONSOR

Imperial College London will act as the Sponsor for this trial. Delegated responsibilities will be assigned to the NHS Trusts taking part in this trial.

This protocol describes the WHEAT trial and provides information about procedures for entering participants. Every care was taken in its drafting, but corrections or amendments may be necessary. These will be circulated to investigators in the trial.

12.5. FUNDING

The United Kingdom Medical Research Council (MRC) are funding this trial. Parents will not be given any financial or material incentive or compensation for enrolling their infants in this trial.

12.6. AUDITS

The trial may be subject to inspection and audit by Imperial College London under their remit as Sponsor and other regulatory bodies to ensure adherence to GCP and the UK Policy Framework for Health and Social Care Research.

For peer review only - http://bmjopen.bmj.com/site/about/guidelines.xhtml

13. TRIAL MANAGEMENT

The trial will be supervised on a day-to-day basis by the Project Management Group (PMG). This group reports to the TSC which is responsible to the trial Sponsor.

The core PMG will consist of Chris Gale (Chief Clinical Investigator), Sena Jawad (Trial Statistician) and NPEU CTU staff including:

- CTU Director
- Senior Trials Managers
- Head of Trials Programming

The Clinical Investigators' Group, (CIG) will meet regularly. This will comprise all members of the co-applicant group and the members of the core PMG.

The trial will be overseen by a Trial Steering Committee (TSC) consisting of an independent chair and at least two other independent members. The Chief Investigator and CTU Director will also sit on the TSC.

A Data Monitoring Committee (DMC) independent of the applicants and of the TSC will review the progress of the trial as agreed and provide advice on the conduct of the trial to the TSC and (via the TSC) to the Sponsor. The DMC will act according to its Charter, which will be agreed at its first meeting.

14. PUBLICATION POLICY

The success of the trial depends on a large number of neonatal health professionals and trials unit staff. Credit for the trial findings will be given to all who have collaborated and participated in the trial including all local coordinators and collaborators, members of the trial committees, the NPEU CTU, and trial staff.

Authorship at the head of the primary results paper will take the form [name], [name]… and [name] on behalf of the WHEAT Trial Collaborative Group, where named authors form part of the writing committee. The writing will be the responsibility of the writing committee which it is anticipated will include all of the investigators. Named authors will be listed in the following order: individual responsible for completing the first draft of the paper, lead analyst, all other members of the writing committee in alphabetical order, lead supervising author. All other contributors to the trial will be listed at the end of the report, with their contribution to the trial identified.

Those responsible for other publications reporting specific aspects of the trial, such as detailed microbiological outcomes, may wish to utilise a different authorship model. Decisions about authorship of additional papers will be discussed and agreed by the trial investigators and the TSC.

Full details of the trial will be made available to parents of infants enrolled in the trial through the trial website: www.npeu.ox.ac.uk/WHEAT.

For peer review only - http://bmjopen.bmj.com/site/about/guidelines.xhtml

15. REFERENCES

1. Battersby C, Longford NT, Mandalia S, Costeloe K, Modi N. Incidence and enteral feed antecedents of severe neonatal necrotising enterocolitis across neonatal networks in England, 2012–13: a whole-population surveillance study. The Lancet Gastroenterology & Hepatology. 2017;2(1):43-51.

2. Duro D, Kalish LA, Johnston P, Jaksic T, McCarthy M, Martin C, et al. Risk factors for intestinal failure in infants with necrotizing enterocolitis: a Glaser Pediatric Research Network study. J Pediatr. 2010;157(2):203-8 e1.

3. Hintz SR, Kendrick DE, Stoll BJ, Vohr BR, Fanaroff AA, Donovan EF, et al. Neurodevelopmental and growth outcomes of extremely low birth weight infants after necrotizing enterocolitis. Pediatrics. 2005;115(3):696-703.

4. Rees CM, Pierro A, Eaton S. Neurodevelopmental outcomes of neonates with medically and surgically treated necrotizing enterocolitis. Arch Dis Child Fetal Neonatal Ed. 2007;92(3):F193-8.

5. Seges RA, Kenny A, Bird GW, Wingham J, Baals H, Stauffer UG. Pediatric surgical patients with severe anaerobic infection: report of 16 T-antigen positive cases and possible hazards of blood transfusion. Journal of Pediatric Surgery. 1981;16(6):905-10.

6. Stritzke AI, Smyth J, Synnes A, Lee SK, Shah PS. Transfusion-associated necrotising enterocolitis in neonates. Arch Dis Child Fetal Neonatal Ed. 2013;98(1):F10-4.

7. Blau J, Calo JM, Dozor D, Sutton M, Alpan G, La Gamma EF. Transfusion-related acute gut injury: necrotizing enterocolitis in very low birth weight neonates after packed red blood cell transfusion. J Pediatr. 2011;158(3):403-9.

8. Paul DA, Mackley A, Novitsky A, Zhao Y, Brooks A, Locke RG. Increased odds of necrotizing enterocolitis after transfusion of red blood cells in premature infants. Pediatrics. 2011;127(4):635-41.

9. Mally P, Golombek SG, Mishra R, Nigam S, Mohandas K, Depalhma H, et al. Association of necrotizing enterocolitis with elective packed red blood cell transfusions in stable, growing, premature neonates. Am J Perinatol. 2006;23(8):451-8.

10. Cunningham KE, Okolo FC, Baker R, Mollen KP, Good M. Red blood cell transfusion in premature infants leads to worse necrotizing enterocolitis outcomes. J Surg Res. 2017;213:158-65.

11. Grave GD, Nelson SA, Walker WA, Moss RL, Dvorak B, Hamilton FA, et al. New therapies and preventive approaches for necrotizing enterocolitis: Report of a research planning workshop. Pediatric Research. 2007;62(4):510-4.

12. Sodhi CP, Neal MD, Siggers R, Sho S, Ma CR, Branca MF, et al. Intestinal Epithelial Toll-Like Receptor 4 Regulates Goblet Cell Development and Is Required for Necrotizing Enterocolitis in Mice. Gastroenterology. 2012;143(3):708-U234.

13. Gordon PV, Swanson JR. Necrotizing enterocolitis is one disease with many origins and potential means of prevention. Pathophysiology: the official journal of the International Society for Pathophysiology / ISP. 2014;21(1):13-9.

For peer review only - http://bmjopen.bmj.com/site/about/guidelines.xhtml

14. Martinussen M, Brubakk A, Torstein V, Yao A. Mesenteric blood flow velocity and its relation to transitional circulatory adaptation in appropriate for gestational age preterm infants. Pediatr Res. 1996;39:275-80.

15. Van Bel F, Van Zwieten P, Guit G, Schipper J. Superior mesenteric artery blood flow velocity and estimated volume feed: duplex Doppler US study of preterm and term neonates. Radiology. 1990;174:165-9.

16. Kempley ST, Gamsu HR. Superior mesenteric artery blood flow velocity in necrotising enterocolitis. Arch Dis Child. 1992;67:793-6.

17. Krimmel GA, Baker R, Yanowitz TD. Blood transfusion alters the superior mesenteric artery blood flow velocity response to feeding in premature infants. Am J Perinatol. 2009;26(2):99-105.

18. Nair J, Gugino SF, Nielsen LC, Allen C, Russell JA, Mathew B, et al. Packed red cell transfusions alter mesenteric arterial reactivity and nitric oxide pathway in preterm lambs. Pediatr Res. 2013;74(6):652-7.

19. Szabo JS, Mayfield SR, Oh W, Stonestreet BS. Postprandial gastrointestinal blood flow and oxygen consumption: effects of hypoxemia in neonatal piglets. Pediatr Res. 1987;21(1):93-8.

20. La Gamma EF, Blau J. Transfusion-related acute gut injury: feeding, flora, flow, and barrier defense. Semin Perinatol. 2012;36(4):294-305.

21. Marin T, Josephson CD, Kosmetatos N, Higgins M, Moore JE. Feeding Preterm Infants during Red Blood Cell Transfusion Is Associated with a Decline in Postprandial Mesenteric Oxygenation. J Pediatr. 2014.

22. El-Dib M, Narang S, Lee E, Massaro AN, Aly H. Red blood cell transfusion, feeding and necrotizing enterocolitis in preterm infants. J Perinatol. 2011;31(3):183-7.

23. Oddie SJ, Young L, McGuire W. Slow advancement of enteral feed volumes to prevent necrotising enterocolitis in very low birth weight infants. Cochrane Database Syst Rev. 2017;8:CD001241.

24. Kirtsman M, Yoon EW, Ojah C, Cieslak Z, Lee SK, Shah PS. Nil-per-os days and necrotizing enterocolitis in extremely preterm infants. Am J Perinatol. 2015;32(8):785-94.

25. Kirpalani H, Whyte RK, Andersen C, Asztalos EV, Heddle N, Blajchman MA, et al. The Premature Infants in Need of Transfusion (PINT) study: a randomized, controlled trial of a restrictive (low) versus liberal (high) transfusion threshold for extremely low birth weight infants. J Pediatr. 2006;149(3):301-7.

26. Whyte R, Kirpalani H. Low versus high haemoglobin concentration threshold for blood transfusion for preventing morbidity and mortality in very low birth weight infants. Cochrane Database Syst Rev. 2011(11):CD000512.

27. Keir A, Pal S, Trivella M, Lieberman L, Callum J, Shehata N, et al. Adverse effects of red blood cell transfusions in neonates: a systematic review and meta-analysis. Transfusion. 2016;56(11):2773-80.

For peer review only - http://bmjopen.bmj.com/site/about/guidelines.xhtml

28. Jasani B, Rao S, Patole S. Withholding Feeds and Transfusion-Associated Necrotizing Enterocolitis in Preterm Infants: A Systematic Review. Adv Nutr. 2017;8(5):764-9.

29. Bell MJ, Ternberg JL, Feigin RD, Keating JP, Marshall R, Barton L, et al. Neonatal necrotizing enterocolitis. Therapeutic decisions based upon clinical staging. Annals of surgery. 1978;187(1):1-7.

30. Parige R, Turner C, Sundaram S, Power S. Enteral feeding during packed red blood cell transfusion in English neonatal units. Arch Dis Child Fetal Neonatal Ed. 2013.

31. Calo JM, Blau J, La Gamma EF, editors. A Survey of Attending Neonatologists on Transfusion Related Acute Gut Injury (TRAGI): The Association between Necrotizing Enterocolitis (NEC) and PRBC Transfusion. Pediatric Academic Societies; 2009: E-PAS.

32. Duley L, Uhm S, Oliver S, Preterm Birth Priority Setting Partnership Steering G. Top 15 UK research priorities for preterm birth. Lancet. 2014;383(9934):2041-2.

33. Bolton-Maggs PHB, Cohen H, Serious Hazards of Transfusion (SHOT) Steering Group. Publisher: Serious Hazards of Transfusion (SHOT). Annual Serious Hazards of Transfusion (SHOT) Report 2012.

34. Bolton-Maggs PHB, Poles D, Watt A, Thomas D, Serious Hazards of Transfusion (SHOT) Steering Group. Publisher: Serious Hazards of Transfusion (SHOT). Annual Serious Hazards of Transfusion (SHOT) Report 2014.

35. Jackups R, Jr., Savage W. Gaps in Research on Adverse Events to Transfusion in Pediatrics. Transfus Med Rev. 2016;30(4):209-12.

36. Keir AK, Wilkinson D. Question 1 Do feeding practices during transfusion influence the risk of developing necrotising enterocolitis in preterm infants? Arch Dis Child. 2013;98(5):386-8.

37. Amin SC, Remon JI, Subbarao GC, Maheshwari A. Association between red cell transfusions and necrotizing enterocolitis. J Matern Fetal Neonatal Med. 2012;25(Suppl 5):85-9.

38. Agwu JC, Narchi H. In a preterm infant, does blood transfusion increase the risk of necrotizing enterocolitis? Arch Dis Child. 2005;90(1):102-3.

39. Derienzo C, Smith PB, Tanaka D, Bandarenko N, Campbell ML, Herman A, et al. Feeding practices and other risk factors for developing transfusion-associated necrotizing enterocolitis. Early Hum Dev. 2014.

40. Luton A. Transfusion-associated necrotizing enterocolitis: translating knowledge into nursing practice. Neonatal network: NN. 2013;32(3):167-74.

41. Marin T, Strickland OL. Transfusion-related necrotizing enterocolitis: a conceptual framework. Advances in neonatal care: official journal of the National Association of Neonatal Nurses. 2013;13(3):166-74.

42. Del Vecchio A, Henry E, D'Amato G, Cannuscio A, Corriero L, Motta M, et al. Instituting a program to reduce the erythrocyte transfusion rate was accompanied by reductions in the incidence of bronchopulmonary dysplasia, retinopathy of prematurity and necrotizing enterocolitis. J Matern Fetal Neonatal Med. 2013;26 Suppl 2:77-9.

For peer review only - http://bmjopen.bmj.com/site/about/guidelines.xhtml

43. Perciaccante JV, Young TE, editors. Necrotizing Enterocolitis Associated with Packed Red Blood Cell Transfusions in Premature Neonates. Pediatric Academic Societies; 2008.

44. ClinicalTrials.gov. Impact of Feeding on Pro-Inflammatory Cytokine Response in Neonates Receiving a RBC Transfusion 2014 [NCT01949896:[Available from: http://clinicaltrials.gov/ct2/show/NCT01949896?term=transfusion+AND+%22necrotising+enterocolitis%22&rank=5.

45. Bode S, Dreyer M, Greisen G. Gastric emptying and small intestinal transit time in preterm infants: a scintigraphic method. J Pediatr Gastroenterol Nutr. 2004;39(4):378-82.

46. Gupta S, Wyllie JP, Plews D, editors. Hemodynamic Effects of Packed Red Blood Cell Transfusion Volume in Premature Infants: Results of a Randomised Controlled Trial. Pediatric Academic Societies; 2007.

47. Battersby C, Longford N, Costeloe K, Modi N, United Kingdom Neonatal Collaborative Necrotising Enterocolitis Study Group. Development of a Gestational Age-Specific Case Definition for Neonatal Necrotizing Enterocolitis. JAMA Pediatr. 2017.

48. Bernardo J, Nowacki A, Martin R, Fanaroff JM, Hibbs AM. Multiples and parents of multiples prefer same arm randomization of siblings in neonatal trials. J Perinatol. 2015;35(3):208-13.

For peer review only

For peer review only - http://bmjopen.bmj.com/site/about/guidelines.xhtml

RECORD OF CHANGES

Version Stage	Versions No	Version Date	Protocol updated & finalised by	Detail the reason(s) for the protocol update
V2.0 26.07.18	V 1.0	24.04.18	PMG	Amendment 1 (substantial):<br>Change of secondary clinical end points from weight (for gestational age at NICU discharge) to growth (change in weight and head circumference for gestational age between birth and NICU discharge).<br>Additional text clarifying collection of pre-transfusion haemoglobin levels (section 5.2 concomitant care)<br>Changes to source data verification (SDV) percentages (section 6.1). Changing review of NEC cases from 10% of cases to all cases and removing SDV on 5% of cases where NEC is not reported.<br>Clarification that there are no upper limits for consent and enrolment<br>Clarification on why no form sample size calculations are conducted and removing details about formal hypothesis testing for clinical outcomes.<br>Adding ISRCTN number<br>Formatting text throughout document

For peer review only

For peer review only - http://bmjopen.bmj.com/site/about/guidelines.xhtml

APPENDIX 1 - Summary of investigations, treatment and assessments

	PERIOD					
	Enrolment	Allocation	Post-allocation			Close-out
**TIMEPOINT**	**After birth, before allocation**	**0**	**Transfus-ion 1 (t_1)**	**t_2**	**t_3, etc.**	**Discharge from neonatal unit**
**ENROLMENT**						
Eligibility screen	X					
Informed "opt-out" consent	X					
Allocation		X				
**COMPARATOR PATHWAYS OF CARE**						
Withholding feeds			X	X	X	
Continuing feeds						
**ASSESSMENTS**						
Baseline variables	Collected from routine data extracted by the NNRD – no involvement of participant					
Outcome variables	Collected from routine data extracted by the NNRD – no involvement of participant					
Other variables	Collected from routine data extracted by the NNRD – no involvement of participant					

For peer review only - http://bmjopen.bmj.com/site/about/guidelines.xhtml

SPIRIT
STANDARD PROTOCOL ITEMS: RECOMMENDATIONS FOR INTERVENTIONAL TRIALS

SPIRIT 2013 Checklist: Recommended items to address in a clinical trial protocol and related documents*

Section/item	Item No	Description	Page
**Administrative information**			
Title	1	Descriptive title identifying the study design, population, interventions, and, if applicable, trial acronym	1
Trial registration	2a	Trial identifier and registry name. If not yet registered, name of intended registry	6, 19
	2b	All items from the World Health Organization Trial Registration Data Set	6, 19
Protocol version	3	Date and version identifier	NA
Funding	4	Sources and types of financial, material, and other support	23-24
Roles and responsibilities	5a	Names, affiliations, and roles of protocol contributors	1-3
	5b	Name and contact information for the trial sponsor	NA
	5c	Role of study sponsor and funders, if any, in study design; collection, management, analysis, and interpretation of data; writing of the report; and the decision to submit the report for publication, including whether they will have ultimate authority over any of these activities	23-24
	5d	Composition, roles, and responsibilities of the coordinating centre, steering committee, endpoint adjudication committee, data management team, and other individuals or groups overseeing the trial, if applicable (see Item 21a for data monitoring committee)	18
**Introduction**			
Background and rationale	6a	Description of research question and justification for undertaking the trial, including summary of relevant studies (published and unpublished) examining benefits and harms for each intervention	8-9
	6b	Explanation for choice of comparators	11-12

For peer review only

Objectives	7	Specific objectives or hypotheses	10
Trial design	8	Description of trial design including type of trial (eg, parallel group, crossover, factorial, single group), allocation ratio, and framework (eg, superiority, equivalence, noninferiority, exploratory)	10

Methods: Participants, interventions, and outcomes

Study setting	9	Description of study settings (eg, community clinic, academic hospital) and list of countries where data will be collected. Reference to where list of study sites can be obtained	11
Eligibility criteria	10	Inclusion and exclusion criteria for participants. If applicable, eligibility criteria for study centres and individuals who will perform the interventions (eg, surgeons, psychotherapists)	11
Interventions	11a	Interventions for each group with sufficient detail to allow replication, including how and when they will be administered	11-12
	11b	Criteria for discontinuing or modifying allocated interventions for a given trial participant (eg, drug dose change in response to harms, participant request, or improving/worsening disease)	12
	11c	Strategies to improve adherence to intervention protocols, and any procedures for monitoring adherence (eg, drug tablet return, laboratory tests)	12
	11d	Relevant concomitant care and interventions that are permitted or prohibited during the trial	12
Outcomes	12	Primary, secondary, and other outcomes, including the specific measurement variable (eg, systolic blood pressure), analysis metric (eg, change from baseline, final value, time to event), method of aggregation (eg, median, proportion), and time point for each outcome. Explanation of the clinical relevance of chosen efficacy and harm outcomes is strongly recommended	12-13
Participant timeline	13	Time schedule of enrolment, interventions (including any run-ins and washouts), assessments, and visits for participants. A schematic diagram is highly recommended (see Figure)	12
Sample size	14	Estimated number of participants needed to achieve study objectives and how it was determined, including clinical and statistical assumptions supporting any sample size calculations	14
Recruitment	15	Strategies for achieving adequate participant enrolment to reach target sample size	14-15

For peer review only - http://bmjopen.bmj.com/site/about/guidelines.xhtml

Methods: Assignment of interventions (for controlled trials)

Allocation:

Sequence generation	16a	Method of generating the allocation sequence (eg, computer-generated random numbers), and list of any factors for stratification. To reduce predictability of a random sequence, details of any planned restriction (eg, blocking) should be provided in a separate document that is unavailable to those who enrol participants or assign interventions	16
Allocation concealment mechanism	16b	Mechanism of implementing the allocation sequence (eg, central telephone; sequentially numbered, opaque, sealed envelopes), describing any steps to conceal the sequence until interventions are assigned	15-16
Implementation	16c	Who will generate the allocation sequence, who will enrol participants, and who will assign participants to interventions	15-16
Blinding (masking)	17a	Who will be blinded after assignment to interventions (eg, trial participants, care providers, outcome assessors, data analysts), and how	16
	17b	If blinded, circumstances under which unblinding is permissible, and procedure for revealing a participant's allocated intervention during the trial	NA

Methods: Data collection, management, and analysis

Data collection methods	18a	Plans for assessment and collection of outcome, baseline, and other trial data, including any related processes to promote data quality (eg, duplicate measurements, training of assessors) and a description of study instruments (eg, questionnaires, laboratory tests) along with their reliability and validity, if known. Reference to where data collection forms can be found, if not in the protocol	14-15
	18b	Plans to promote participant retention and complete follow-up, including list of any outcome data to be collected for participants who discontinue or deviate from intervention protocols	14-15
Data management	19	Plans for data entry, coding, security, and storage, including any related processes to promote data quality (eg, double data entry; range checks for data values). Reference to where details of data management procedures can be found, if not in the protocol	14-15
Statistical methods	20a	Statistical methods for analysing primary and secondary outcomes. Reference to where other details of the statistical analysis plan can be found, if not in the protocol	16-18

For peer review only - http://bmjopen.bmj.com/site/about/guidelines.xhtml

	20b	Methods for any additional analyses (eg, subgroup and adjusted analyses)	16-18
	20c	Definition of analysis population relating to protocol non-adherence (eg, as randomised analysis), and any statistical methods to handle missing data (eg, multiple imputation)	16-18
**Methods: Monitoring**			
Data monitoring	21a	Composition of data monitoring committee (DMC); summary of its role and reporting structure; statement of whether it is independent from the sponsor and competing interests; and reference to where further details about its charter can be found, if not in the protocol. Alternatively, an explanation of why a DMC is not needed	18
	21b	Description of any interim analyses and stopping guidelines, including who will have access to these interim results and make the final decision to terminate the trial	18
Harms	22	Plans for collecting, assessing, reporting, and managing solicited and spontaneously reported adverse events and other unintended effects of trial interventions or trial conduct	18-19
Auditing	23	Frequency and procedures for auditing trial conduct, if any, and whether the process will be independent from investigators and the sponsor	15
**Ethics and dissemination**			
Research ethics approval	24	Plans for seeking research ethics committee/institutional review board (REC/IRB) approval	19
Protocol amendments	25	Plans for communicating important protocol modifications (eg, changes to eligibility criteria, outcomes, analyses) to relevant parties (eg, investigators, REC/IRBs, trial participants, trial registries, journals, regulators)	17-18
Consent or assent	26a	Who will obtain informed consent or assent from potential trial participants or authorised surrogates, and how (see Item 32)	19
	26b	Additional consent provisions for collection and use of participant data and biological specimens in ancillary studies, if applicable	19
Confidentiality	27	How personal information about potential and enrolled participants will be collected, shared, and maintained in order to protect confidentiality before, during, and after the trial	14-15
Declaration of interests	28	Financial and other competing interests for principal investigators for the overall trial and each study site	23

view only

Access to data	29	Statement of who will have access to the final trial dataset, and disclosure of contractual agreements that limit such access for investigators	23
Ancillary and post-trial care	30	Provisions, if any, for ancillary and post-trial care, and for compensation to those who suffer harm from trial participation	NA
Dissemination policy	31a	Plans for investigators and sponsor to communicate trial results to participants, healthcare professionals, the public, and other relevant groups (eg, via publication, reporting in results databases, or other data sharing arrangements), including any publication restrictions	19-20
	31b	Authorship eligibility guidelines and any intended use of professional writers	24
	31c	Plans, if any, for granting public access to the full protocol, participant-level dataset, and statistical code	NA
**Appendices**			
Informed consent materials	32	Model consent form and other related documentation given to participants and authorised surrogates	NA
Biological specimens	33	Plans for collection, laboratory evaluation, and storage of biological specimens for genetic or molecular analysis in the current trial and for future use in ancillary studies, if applicable	NA

*It is strongly recommended that this checklist be read in conjunction with the SPIRIT 2013 Explanation & Elaboration for important clarification on the items. Amendments to the protocol should be tracked and dated. The SPIRIT checklist is copyrighted by the SPIRIT Group under the Creative Commons "Attribution-NonCommercial-NoDerivs 3.0 Unported" license.

For peer review only
